# Predictors of multiple sclerosis progression: A systematic review of conventional magnetic resonance imaging studies

**DOI:** 10.1371/journal.pone.0300415

**Published:** 2024-04-16

**Authors:** Nima Broomand Lomer, Kamal AmirAshjei Asalemi, Alia Saberi, Kasra Sarlak

**Affiliations:** 1 Faculty of Medicine, Guilan University of Medical Sciences, Rasht, Iran; 2 Department of Neurology, Poursina Hospital, Faculty of Medicine, Guilan University of Medical Sciences, Rasht, Iran; University of Bahrain, BAHRAIN

## Abstract

**Introduction:**

Multiple Sclerosis (MS) is a chronic neurodegenerative disorder that affects the central nervous system (CNS) and results in progressive clinical disability and cognitive decline. Currently, there are no specific imaging parameters available for the prediction of longitudinal disability in MS patients. Magnetic resonance imaging (MRI) has linked imaging anomalies to clinical and cognitive deficits in MS. In this study, we aimed to evaluate the effectiveness of MRI in predicting disability, clinical progression, and cognitive decline in MS.

**Methods:**

In this study, according to PRISMA guidelines, we comprehensively searched the Web of Science, PubMed, and Embase databases to identify pertinent articles that employed conventional MRI in the context of Relapsing-Remitting and progressive forms of MS. Following a rigorous screening process, studies that met the predefined inclusion criteria were selected for data extraction and evaluated for potential sources of bias.

**Results:**

A total of 3028 records were retrieved from database searching. After a rigorous screening, 53 records met the criteria and were included in this study. Lesions and alterations in CNS structures like white matter, gray matter, corpus callosum, thalamus, and spinal cord, may be used to anticipate disability progression. Several prognostic factors associated with the progression of MS, including presence of cortical lesions, changes in gray matter volume, whole brain atrophy, the corpus callosum index, alterations in thalamic volume, and lesions or alterations in cross-sectional area of the spinal cord. For cognitive impairment in MS patients, reliable predictors include cortical gray matter volume, brain atrophy, lesion characteristics (T2-lesion load, temporal, frontal, and cerebellar lesions), white matter lesion volume, thalamic volume, and corpus callosum density.

**Conclusion:**

This study indicates that MRI can be used to predict the cognitive decline, disability progression, and disease progression in MS patients over time.

## Introduction

Multiple Sclerosis (MS) is a chronic autoimmune disease that affects the central nervous system (CNS) and leads to demyelination, axonal loss, and neurodegeneration. The disease is caused by a complex interaction of environmental and genetic factors that are not yet fully understood [1, 2]. MS presents with a wide range of symptoms including sensory disturbances, walking difficulties, vision problems, intestinal and urinary dysfunction, cognitive and emotional impairment, dizziness, vertigo, sexual problems, speech difficulties, seizures, and headaches [3, 4]. MS is classified into four subgroups based on phenotype: clinically isolated syndrome (CIS), relapsing-remitting MS (RRMS), secondary-progressive MS (SPMS), and primary-progressive MS (PPMS). RRMS is the most common form of the disease, affecting approximately 85% of patients at presentation. It is characterized by acute exacerbations followed by clinically stable periods [5]. PPMS, on the other hand, presents with a slowly progressive reduction in neurological function from the start without clinical relapses [6, 7]. Naturally, RRMS tends to convert to SPMS which is an irreversible gradual disability progression [8]. In the past, nearly 10% of RRMS patients progressed to SPMS in a 5-year period, 25% in 10 years, and 75% in 30 years. However, with the advent of more treatment options and early diagnosis, the risk of SPMS conversion has decreased to about 2%, 9%, and 27% in a 10-year, 15-year, and 20-year period, respectively [9–11]. In addition to physical disability, impairment of cognitive function is also a common manifestation of MS. Neuropsychological abnormalities are observed in 40–70% of MS patients, and cognitive impairment is a predictor of disease progression [12]. MS in cognitively impaired patients is more likely to progress in upcoming years [13]. The most common cognitive impairments in MS include reduced speed of information processing and working memory, which can disrupt data retention ability and short-term memory [14–17]. Unfortunately, the underlying mechanisms of cognitive impairment in MS are not yet fully understood [18, 19].

Magnetic resonance imaging (MRI) plays a pivotal role in the detection, prognosis, and evaluation of disease activity in MS [20–23]. Focal lesions, atrophies, and normal appearing tissue damages are among the MS pathologies that can be detected using MRI [20]. White matter lesions and deep gray matter atrophy typically arise in the early stages of the disease, while cortical atrophy and demyelination emerge in later stages [24–27].

Features of MS lesions in the brain or spinal cord, including the presence of lesions or changes in the size of certain CNS structures such as the thalamus, corpus callosum, cerebellum, limbic system, and spinal cord are not addressed in the latest version of McDonald Criteria (2017) [23] or in recent guidelines for determining disease progression or deciding for escalation or change of treatment in MS disease. Considering this, here we aimed to evaluate the potential of conventional MRI markers in predicting clinical disability, disease progression, and cognitive decline in MS patients.

## Methods and materials

### Eligibility criteria

We included studies with the following criteria: [1] Definite diagnosis of MS based on the revised McDonald’s criteria of 2017 [2, 23], Applied conventional MRI, and [3] Focused on evaluating the progression of disability or cognitive decline in MS patients. To ensure the quality of the data, we excluded various types of publications, including review articles, animal studies, letters and commentaries, case reports, case series, book chapters, conference abstracts, and non-English studies. Furthermore, the study only included research conducted among patients with RRMS or progressive forms of the disease, while studies conducted among patients with CIS were excluded. We excluded studies with the usage of AI (Deep learning and Machine learning methods) in the prediction of course of disease.

### Search strategy

We conducted this systematic review according to the guideline of preferred reporting items for Systematic reviews and Meta-Analysis (PRISMA) [28]. Search was performed in PubMed, Embase and Web of Science databases from 2010 until July 2023 to identify the relevant studies using the keywords below:

*("progressive multiple sclerosis" OR "Multiple Sclerosis*, *Chronic Progressive"[Mesh] OR "progressive MS" OR "primary progressive multiple sclerosis" OR "secondary progressive multiple sclerosis" OR "primary progressive MS" OR "secondary progressive MS" OR PPMS OR SPMS) AND ("relapsing remitting multiple sclerosis" OR "relapsing-remitting multiple sclerosis" OR "relapsing-remitting MS" OR "relapsing/remitting multiple sclerosis" OR "relapsing/remitting MS" OR "Multiple Sclerosis*, *Relapsing-Remitting"[Mesh] OR "relapsing-remitting MS" OR"relapse-onset MS" OR "relapse*-*onset multiple sclerosis" OR RRMS) AND (MRI OR "magnetic resonance imaging" OR "magnetic resonance imaging"[MeSH] OR imaging) AND (2010*:*2023[pdat])*

**Fig 1:**
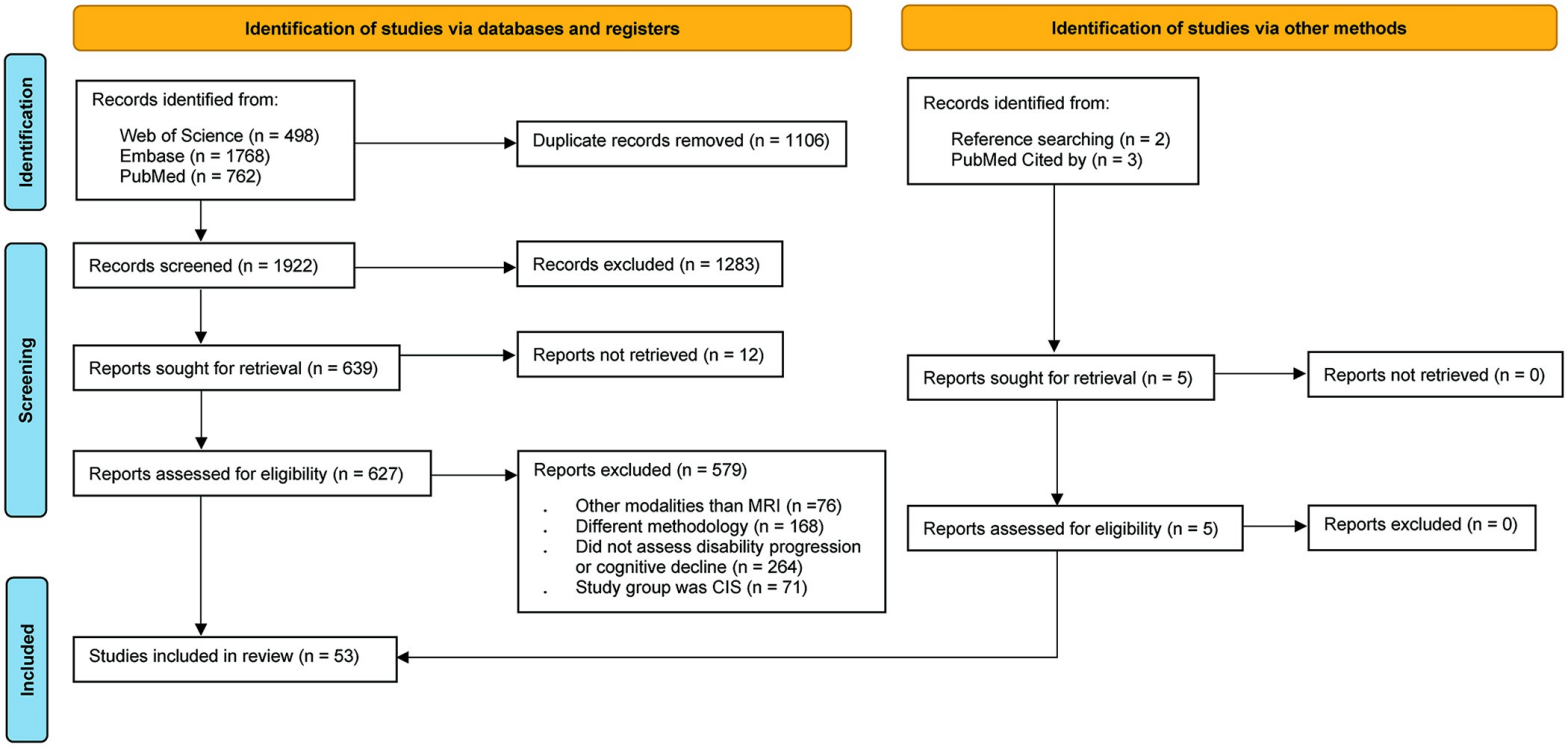
PRISMA flow diagram of database searching and study selection.

We made a slight adjustment to our search strategy to integrate with two other databases. Initially, there were no restrictions on the type of studies, their location, or language. We screened and extracted data from all studies conducted from 2010 to July 2023 using EndNote software [29]. Flow diagram of the database searching and study selection according to the PRISMA guideline is presented below in Fig 1.

### Screening and data extraction

This stage was conducted in three distinct phases by two independent authors, namely K.A.A and K.S. In the first phase, the titles and abstracts of the records were carefully screened to determine their initial eligibility for inclusion in the study. In the event of any discrepancies, the third and fourth authors, N.B.L and A.S, were consulted to resolve the issue by consensus. In the second phase, the full text of the selected records was retrieved. Only those articles that specifically studied MRI markers in relation to disability progression or cognitive decline in RRMS or progressive forms of MS were included. In the third and final phase, relevant data was extracted and recorded in a data collection table, which included important information such as the demographic features of the study participants (year of study, number and studied groups of participants, mean age, and disease duration), the imaging methodology used (field strengths in Tesla and studied parameter), and the correlations of MRI markers with disability progression and cognitive decline.

### Data items

In this review, we aimed to assess any correlations of MRI markers with disability progression and cognitive decline in RRMS and progressive forms of MS. These terms are defined as follows:

**Disability:** Disability progression in MS is a broad term referring to the worsening of physical, cognitive and emotional symptoms during the disease course. We mainly aimed at the physical disability mostly measured by Expanded Disability Status Scale (EDSS) among included studies. Depending on the affected area of CNS, physical disability progression can manifest with different symptoms including muscle weakness, balance and coordination problems, fatigue, tremors and difficulty walking. In addition, Timed 25-Foot Walk (T25FWT) and 9-Hole Peg Test (9HPT) can be utilized to assess disability outcomes. The T25FWT assesses an individual’s time to walk 25 feet as swiftly as possible while ensuring safety. Prolonged completion times indicate increased disability levels. Meanwhile, the 9HPT evaluates arm and hand functionality, employing a small container with nine holes and pegs. Participants are instructed to place and remove the pegs from the holes individually as rapidly as possible. Longer completion times signify higher disability.

**Progression:** The progression of MS refers to how the disease evolves and advances over time. CIS and RRMS phenotypes tend to progress and convert to the progressive phenotypes of MS.

**Cognition:** Cognitive decline, a representative of disability progression in MS, refers to the progressive deterioration of cognitive functions, including memory, attention, information processing speed, executive functions, and problem-solving abilities. It can significantly impact daily functioning, work performance, and overall quality of life. Two commonly used cognitive function assessment tools in studies are the Symbol Digit Modalities Test (SDMT) and the Paced Auditory Serial Addition Test (PASAT). The SDMT evaluates processing speed and attention by matching symbols with numbers within a time limit, while the PASAT measures processing speed, flexibility, and working memory by requiring participants to add orally presented numbers in sequence.

### Risk of bias assessment

In accordance with the PRISMA guidelines [28], the quality assessment of the studies included in this systematic review was conducted using the Joanna Briggs Institute Critical Appraisal tools (JBI) specifically designed for cross-sectional studies [30]. Two independent authors (N.B.L and K.S) conducted the assessment by answering 11 questions that evaluate different domains of the studies to ascertain their potential risk of bias. The questions could be answered with ‘yes’, ‘no’ or ‘unclear’. Any discrepancies between the two reviewers were discussed and resolved by achieving a consensus. The risk of bias for each individual study was determined based on the following criteria: low risk of bias if 70% of answers scored yes, moderate risk if 50 to 69% questions scored yes and high risk of bias if yes scores were below 49% [31].

## Results

A total of 3028 articles were identified after conducting a thorough search of the database, including 762 articles from PubMed, 498 from Web of Science, and 1768 from Embase. Following the elimination of duplicates, 1922 articles remained for title and abstract screening. Subsequently, 639 articles were retrieved for a full text analysis, out of which 12 records were not found. During the comprehensive full text screening, 579 articles were excluded for not meeting the inclusion criteria. An additional five studies were found through other sources and met the inclusion criteria, leading to a total of 53 studies being included in this review.

Correlating MRI markers in cortical lesions and gray matter alterations of MS patients were assessed in fifteen studies, spinal cord alterations in twelve studies, corpus callosum alterations in three studies, cerebellum alterations in six studies, thalamus alterations in eleven studies, limbic system alterations in two studies, lesion atrophy in two studies, whole brain and white matter lesion volume in nineteen studies.

### MRI markers predicting the disability progression

Abnormalities in the gray matter, whether deep or cortical, including atrophy or lesion in the cortex, can predict the progression of disability among patients with MS. Several studies have shown a strong correlation between cortical lesion and EDSS score [32–38] with cortical lesion volume being a predictor of neurologic disability progression during follow-up [35]. Gray matter atrophy has also been identified as a predictor of higher EDSS scores [34, 39, 40]. In addition, the ratio of gray matter (GM) to normal appearing white matter (NAWM) in recently diagnosed RRMS patients can predict disability progression [41]. The deep gray matter has been found to be a predictor of time-to-EDSS progression [42].

In white matter (WM), EDSS score was significantly correlated with WM lesion volume, central atrophy, lesion probability in the periventricular WM at the left frontal horn and around the posterior horns and with whole-brain volume particularly with widths of third and lateral ventricle [32, 37, 43–45]. The presence of confluent lesions (in RRMS), higher number of T2 lesions, lower baseline T2-lesion volume (T2LV), lower normalized brain volume (NBV), higher percentage brain volume change (PBVC) between year 2 and baseline and presence of ≥ 4 slowly expanding lesions (SELs) were defined as prognostic factors for EDSS worsening and disability progression [46–48, 83].

In the corpus callosum, some indices, including corpus callosum index (CCI) and corpus callosum lesion volume (CCLV), which indicate corpus callosum (CC) damage, were associated with disability progression and EDSS change [49, 50].

Thalamic volume change especially in the anterior, ventral anterior, ventral lateral and pulvinar nuclei inversely correlated with EDSS [50–53]. Furthermore, the EDSS was negatively associated with thalamic iron [54].

Spinal cord changes such as atrophy (GM and WM) or lesions were indicators of disability and worsening EDSS. Some studies suggested that smaller cervical cross-sectional area (CS-SCA), especially CSA-C2, loss of spinal cord volume (SCV), baseline annualized percentage upper cervical cord cross-sectional area change (aUCCA), and the number of spinal cord segments affected by T2-lesions are all predictive factors for disability [34, 40, 55–60].

In all CNS structures, atrophied lesion volume was significantly associated with disability progression [61].

### MRI markers predicting the progression of disease

Cortical lesions and gray matter volume are two most significant determinants of a progressive disease [34, 35]. Cortical lesions are more prevalent in SPMS subjects compared to RRMS subjects [35] and higher baseline cortical lesions predicted conversion to SPMS [62]. Temporal gray matter atrophy is faster in SPMS than RRMS [42]. The GM/NAWM ratio is a predictor of SPMS conversion in recently diagnosed RRMS patients, implicating that GM and NAWM are influenced differently regarding disease development since early stages of MS [41].

Some MS lesion characteristics and also atrophy of brain are among other key markers and predictors of MS progression. Notably, iron rims serve as a representation of the chronic active nature of MS lesions, indicating a more severe and damaging form of the disease [63]. In a study, the only longitudinal MRI marker that was capable of distinguishing patients who deteriorated gradually from those who remained stable was brain atrophied T2-LV [64]. In another 9.1-year longitudinal study, the number and volume of T2 hyperintense lesions and lower NBV were significantly associated with conversion to SPMS [47]. Higher annualized percentage ventricular volume change (aPVVC) during the first 2 years was observed in patients with progressive disease compared to patients with no progression [65]. Central atrophy was associated with disease progression over 5.5 years in early RRMS [45]. One study showed that significant discriminative MRI atrophy measurements in RRMS vs SPMS are as followed: Index of frontal atrophy, Index of EVANS, Huckman Index, Bicaudatus Index and Width of third ventricle. For differentiating RRMS from SPMS; Remission-Progression Index formula can be used [66]: Remission-Progression Index = (RAVLT 1–5 SUM + DSST)/Huckman Index.

The corpus callosum index is an important prognostic factor for the progression of MS. It has been observed that individuals diagnosed with SPMS exhibit lower levels of CCI at the time of diagnosis, while also experiencing a greater decline in annual CCI compared to those with RRMS [67].

Deeper nuclei impairment, higher thalamic lesion volume and higher thalamic volume reduction was seen in SPMS compared with the RRMS group [38, 51, 68]. Baseline volume and the rate of annual volume loss of the ventral lateral nucleus were significant predicting factors of disease progression [53].

Spinal cord abnormalities including atrophy and lesion and gadolinium-enhancement at disease onset and during disease are also predictors of MS progression and conversion to progressive forms [69]. SCV loss particularly cervical GM atrophy is a predicting factor for progression. Although, cervical CS-SCA, especially cross-sectional area of C2 (CSA-C2) is significantly smaller in PMS compared to RRMS, but thoracic SCAs are not significantly different between types of MS [55–57, 60]. Reduction of UCCA over 24 months is seen on all MS types and is higher in SPMS [59]. Patients who develop SPMS exhibit accelerated cord atrophy rates before conversion and decelerated rates after conversion [70]. Clusters of cord atrophy are mainly found in the lateral and posterior cord segments [71].

Atrophy of the cerebellum, especially cerebellar posterior superior lobe atrophy was higher in SPMS compared to RRMS [72–74]. Significantly higher volumes and numbers of cerebellar cortical lesions were found in SPMS and PPMS compared to RRMS and CIS [75, 76]. Although these changes manifest during the progression of the disease and may not be immediately apparent at the onset of the condition, hence they may not be regarded as reliable predictors of disease progression.

### MRI markers predicting the cognitive decline

Cortical gray matter volume was an MRI predictor of cognitive decline [77]. But cortical lesion (CL) volume and CL load were not significant predictors of neuropsychological outcomes, and were only associated with impairing the more challenging cognitive tests such as Trail Making Test (TMT-B) [32, 33].

Brain atrophy was correlated with verbal memory impairment and other neurocognitive symptoms. Third ventricle width and bicaudatus ratio correlated mostly with the performed cognitive tests particularly Symbol Digit Modalities Test (SDMT) [66]. In RRMS, atrophy of WM was correlated with verbal memory performances [78].

Some MS lesion characteristics were predictors of cognitive impairment. Atrophied T2-LV among PMS patients, was related to follow-up SDMT of cognitive tests [64]. T2-lesion load (T2-LL) was recognized as an important predictor of memory function, cognitive efficiency and overall cognition [79]. Temporal, frontal and cerebellar hemispherical lesions had correlations with SDMT test performance, and a small cluster in left parietal with SDT. Inability of keeping recently learned information in memory was found to be correlated with lesions in superior parietal and left frontopolar and with adjacent regions of amygdalae and hippocampus [43]. White matter lesion volume (WMLV) was more strongly correlated with the cognitive tests (Paced Auditory Serial Addition Test (PASAT) and SDMT) compared to CL volume [32].

Decrease of thalamic volume was seen with a decrease in cognitive performance [52]. Normalized thalamic volume and anterior thalamic radiation integrity were among the predictors of cognitive decline [77, 79]. Both verbal and written parts of the SDMT test indicated moderate to strong correlations with the volume of thalamus nuclear groups [51].

In a study, CC density was another independent predictor of brief visuospatial memory test (BMVT) [49].

In RRMS, verbal memory performances correlated with atrophy of WM and left hippocampus [78]. Worse SDMT scores correlated with smaller normalized volume of the hippocampus and amygdala of each hemisphere and reduced R2t of the right hippocampus and amygdala, while worse performance on the 2s PASAT correlated with reduced R2t of the left amygdala [80]. The aforementioned alterations are indicative of cognitive impairment and therefore warrant the attention of medical professionals to evaluate potential cognitive decline in patients. However, it is important to note that these changes cannot be deemed as absolute predictors of cognitive impairment.

Lower cerebellar volumes, prominently posterior superior lobe (VI + Crus I) correlated with scores of SDMT and PASAT [72]. But these changes occurred in parallel with cognitive impairment and cannot predict it. An overview of included studies is shown in Table 1. We tried to analyze Table 1 with an aim to classify the results according to the region assessed, which ranged from cortical and gray matter, spinal cord, corpus callosum, cerebellum, thalamus, limbic system, lesion atrophy, brain volumetry, to lesions and white matter.

**Table 1:** An overview of the literature regarding the studies with correlations of MRI markers with disability progression, progression of the disease and cognitive decline in studies participants.

Study	Demographic Features			Imaging Methodology		Correlations with MRI markers
	Participants	Mean Age ± SD (years) or range	Mean Disease Duration ± SD (years) or range	F/S (T)	Parameter Studied	
Papadopoulou et al. 2013—C/S** [32]	65 CIS and RRMS	49.1 ± 1.85	17.4 ± 9.4	1.5 T	Cortical Lesion volume	**Disability:** Correlations between CL volume and EDSS. (r = 0.206; p = 0.051) **Cognition:** Assessment tools: SDMT, PASAT-3.CL volume was not predictor of neuropsychological outcomes. Subgroup analysis in RRMS: CLV only correlated with SDMT (r = -0.301, p = 0.019). In multiple regression analysis: CL load had no associations with any cognitive outcomes.
	26 SPMS and PPMS					
Matsushita et al. 2018—C/S [33]	13 CL group (9 RRMS + 1 PPMS + 3 SPMS)	43.1 ± 10.1	13.8 ± 8.2	3 T	Cortical Lesion	**Disability:** Higher EDSS score in the CL group (2.8 ± 1.8) compared to non-CL group (0.5 ± 0.8); p = 0.009). **Progression:** In the non-CL group, all six patients had RRMS. In the CL group, 9 had RRMS, 1 had PPMS and 3 had SPMS. **Cognition:** No significant differences in the MMSE1 score, RCPM2 score, RCPM time, RBMT3 SPS4, RBMT SS5, TMT6-A, category WF7 task performance, visuospatial construction copying performance, letter WF task performance, visuospatial construction drawing performance and 1-s PASAT8, and 2-s PASAT between the CL and non-CL groups. The z-score analysis of the TMT and PASAT values were indicative of significant performance deterioration in CL group.
	6 non-CL Group (6 RRMS)	46.7 ± 9.4	16.7 ± 12.2			
Haider et al. 2021—C [34]	21 CIS	60.5 ± 7.1	30.7 ± 1	3 T	Cortical Lesion	**Disability:** Highest EDSS scores were detected in 3 RRMS patients with cortical lesions. Cortical lesion counts (β: 0.37, 95% CI: 0.23 to 0.508), cervical spinal cord volume (β: –0.27, 95% CI: –0.421 to– 0.109), grey matter volume (β:– 0.26, 95% CI:– 0.444 to– 0.074) collectively explained 60% (R2) of the variance of the EDSS In the model including only cortical lesions, 43% (R2) of the EDSS could be explained. Atrophy of GM was more predictive for EDSS. **Progression:** 30-year follow-up: the most important differences in MRI markers between SPMS and RRMS patients were the number of cortical lesions (cortical lesions had 88% specificity and 100% sensitivity), and GMV which was lower in SPMS. No cortical lesions detected in CIS, in 3 of 27 RRMS patients and in all SPMS patients.
	27 RRMS	60.6 ± 6.4	30.9 ± 1			
	15 SPMS	61.9 ± 6.7	30.8 ± 0.9			
Treaba et al. 2019—C [35]	20 RRMS	41.3 ± 10.5	6.0 ± 6.2	7 T	Cortical Lesion	**Disability:** Total CLV was identified as an independent predicting factor for baseline EDSS (β = 1.5, P = .001) and EDSS changes (β = 0.5, P = 0.003) at follow-up (near 1.5 years). **Progression:** Cortical lesion accumulation was bigger in SPMS than RRMS (3.6 lesions/year ± 4.2 vs 1.1 lesions/year ± 0.9, respectively; P = 0.03) and preferentially localized in sulci.
	11 SPMS	39.9 ± 8.5	19.9 ± 9.0			
	10 HC	39.9 ± 0.5				
Kalinin et al. 2020—C/S [36]	ICL—Group (15 RRMS, 1 PPMS)	27 (25–36.5) *	15 (9–34.5) months *	3 T	Intracortical lesions	**Disability:** Patients with intracortical lesions had higher EDSS scores (P = 0.02).
	ICL+ Group (39 RRMS, 12 SPMS, 4 PPMS)	40 (32–53) *	77 (26–171) months *			
Calabrese et al. 2010—C/S [37]	76 RRMS	34.2	4.8	1.5 T	Cortical Lesion volume	**Disability:** Baseline CLV correlated with baseline EDSS (r = 0.36, p = 0.001) and EDSS changes (r = 0.51, p = 0.001) over time. In both patient groups, baseline CLV was identified as an independent predicting factor for EDSS worsening and GM volume change at follow-up. **Progression:** Baseline CLs were detected more among SPMS (74.2%) than RRMS (64.4%). During follow-up, 0.8 new CL/patient/year in RRMS and 1.0 new CL/patient/year in SPMS were seen (all non-significant). Increase of CLV and number was significantly higher in the 52 clinically worsened patients compared to those who remained stable.
	31 SPMS	41.1	11.6			
Scalfari et al. 2018—C [62]	160 RRMS remained RRMS	31.5 ± 10.6	7.8 ± 1.3	1.5 T	Cortical lesions number and thickness	**Progression**: Higher baseline cortical lesions significantly correlated with the higher risk of SPMS conversion (hazard ratio of 2.16 for 2 CLs, 4.79 for 5 CLs, and 12.3 for 7 CLs).
	59 RRMS converted to SPMS	34.2 ± 7.6	8.2 ± 1.0			
Louapre et al. 2018—C/S [38]	10 Early RRMS	38.3 ± 10.5	2.8 ± 1.0	3 T—7 T	Cortical lesions volume	**Disability:** EDSS correlated positively with CLV (Adj R² = 30%). **Progression:** CLV was higher in SPMS compared to other subgroups of MS. **Cognition:** CLV was the most powerful, independent predictor, explaining 40% of the variance of SDMT.
	18 RRMS	44.3 ± 7.6	11.4 ± 4.2			
	13 SPMS	45.5 ± 8.1	18.5 ± 8.4			
	17 HC	39.3 ± 8.8	NA			
Pinter et al. 2015—C/S [79]	17 CIS	33.1 ± 9.1	0.4 ± 0.8	3 T	Cortical volume	**Cognition**: In univariate analysis there was a positive effect of NCV (βj = 0.39; p<0.05) on overall cognition. Cognitive efficiency and overall cognitive function were strongly predicted by cortical volume.
	47 RRMS	35.8 ± 10.5	9.0 ± 8.7			
	5 SPMS	41.8 ± 9.3	11.3 ± 6.0			
Eijlers et al. 2018—C [77]	168 Cognitively Stable (144 RRMS, 15 SPMS, 9 PPMS)	46.7 ± 11.0	14.2 ± 8.3	3 T	Cortical gray matter volume	**Cognition**: Cortical GMV was defined as the only significant MRI predictor of cognitive decline in a whole-brain model (Nagelkerke R2 = 0.22, P<0.001). WM integrity damage was predictor of early RRMS cognitive decline, while in late RRMS and progressive MS, it was predicted by cortical atrophy.
	66 Cognitively Declining (38 RRMS, 18 SPMS, 10 PPMS)	49.7 ± 10.8	15.9± 8.4			
	60 HC	46.4 ± 9.9	NA			
Burgetova et al. 2017—C/S [54]	80 RRMS	46.9 ± 7.0	12.4 ± 10.7	1.5 T	Iron in deep gray matter	**Disability**: EDSS score was positively associated with iron accumulation in the putamen of RRMS and PPMS and caudate of RRMS patients.
	40 EDSS matched RRMS	48.6 ± 7.0	13.2 ± 11.0			
	24 PPMS	47.4 ± 6.8	7.7 ± 3.3			
	20 HC	48.0 ± 7.3	NA			
Rocca et al. 2021—C/S [39]	34 CIS	33.8 (19–50)	0.5 (0.08–3)	3 T	Gray matter volume	**Disability**: Lower NGMV (OR = 0.98, 95%CI = 0.96–0.99, p = 0.008) and lower GMV in the cerebellar network (OR = 0.40, 95%CI = 0.19–0.85, p = 0.01) are independent predictors of disability worsening (AUC = 0.83).
	226 RRMS	44.0 (18–70)	12.7 (0.1–37)			
	95 SPMS	54.3 (33–72)	21.5 (3–46)			
	43 PPMS	55.4 (27–77)	15.8 (2–45)			
	170 HC	40.0 (19–75)	NA			
Tsagkas et al. 2021—C [40]	140 RRMS	43.8 ± 10.2	14.0 ± 8.7	1.5 T	Atrophy of gray matter	**Disability**: Higher baseline GM and GM AVCR in SPMS were associated with higher T25FWT deterioration (mean yearly decrease of 1/T25fwt of -4.2 × 10–3±1.7 × 10–4; p = 0.087). Lower baseline GMV in RRMS was associated with higher D9HPT deterioration (mean yearly increase of log [D9HPT] of 0.012 ± 2.0× 10–3, p = 7.5 × 10–7)
	43 SPMS	55.0 ± 8.8	21.3 ± 9.2			
Eshaghi et al. 2018—C [42]	253 CIS	33.0 ± 8.0	0.4 ± 1.4	Various	Cortical gray matter and deep gray matter volume	**Disability:** Time-to-EDSS progression was predicted by DGM (hazard ratio = 0.73, 95% CI: 0.65, 0.82; p<0.001): for every SD decrease in baseline DGMV, there was a 27% higher risk of presenting a shorter EDSS progression time during follow-up. **Progression:** Lowest volumes of DGM and cortical GM at baseline were detected in SPMS. DGM showed the fastest annual rate of atrophy out of all imaging markers, prominently in SPMS (-1.45%) and RRMS (-1.34%) compared to CIS (-0.88%) and HCs (-0.94%) [p<0.01]. Temporal (- 1.21%) and parietal (-1.24%) GM atrophy was fastest in SPMS (All p values <0.05).
	708 RRMS	38.2 ± 9.8	6.7 ± 7.3			
	128 SPMS	48.2 ± 9.8	15.6 ± 9.9			
	125 PPMS	48.5 ± 10.1	6.8 ±5.9			
	203 HC	38.7 ± 10.5	NA			
Moccia et al. 2017—C [41]	119 RRMS remained RRMS	32.7 ± 7.4	4.2 ± 2.8	Various	Gray: White matter ratio	**Disability:** EDSS worsening was associated with GM/ NAWM ratio (coefficient, 2.918; 95%CI, 4.739–1.097). Patients diagnosed with a higher GM/ NAWM ratio had a 90% lower rate of reaching EDSS 4.0 (hazard ratio, 0.111; 95% CI, 0.020–0.609) compared to patients with lower GM/ NAWM ratio. **Progression:** Higher baseline GM/NAWM ratio associated with lower rate of converting to SPMS (hazard ratio, 0.017; 95% CI, 0.001–0.203).
	30 RRMS converted to SPMS	34.2 ± 6.3	4.6 ± 2.7			
Kearney et al. 2016—C/S [81]	25 CIS	36.5 ± 9.0	0.4 ± 0.4	3 T	Spinal Cord Focal lesions (GM & WM)	**Disability:** Lesion number per patient in both the lateral column and expanding to gray matter had independent associations with disability (p < 0.001). **Progression:** Percentage of patients with focal lesions involving at least two WM columns and expanding to gray matter was higher in SPMS compared to RRMS (p = 0.03) and PPMS (p = 0.015). Diffuse abnormalities were more common in both PPMS and SPMS, compared with RRMS (OR 6.1 (p = 0.002) and OR = 5.7 (p = 0.003), respectively).
	35 RRMS	38.7 ± 9.7	6.5 ± 5.2			
	30 PPMS	50.6 ± 9.9	10.4 ± 7.5			
	30 SPMS	51.1 ± 9.2	19.9 ± 11.5			
Kantarci et al. 2016—C [82]	324 RIS remained RRMS	38.6 (14–74) *	NA	Various	Spinal cord lesions	**Progression**: PPMS cases had more SC lesions (100%) than CIS/MS cases (64%) and asymptomatic cases (23%) within the follow-up period (P = 0.005).
	113 RIS converted to CIS/M	32 (11–70) *	NA			
	15 RIS converted to PPMS	43.3 (20–66) *	NA			
Nakamura et al. 2020—C/S [55]	111 RRMS	43.9 ± 12.7	13.8 ± 10.1	1.5 T- 3 T	Spinal cord area	**Disability:** CS-SCA at all four levels was negatively correlated with EDSS scores (P < 0.0001 at C2/C3 and C3/C4, P = 0.0002 at T8/T9, and P = 0.002 at T9/T10). And FSS for pyramidal symptoms (P = 0.0002 at C2/C3, P < 0.0001 at C3/C4 and T8/T9, and P = 0.0005 at T9/T10). Cervical CS-SCA was more strongly correlated with EDSS compared to thoracic CS-SCA. **Progression:** Cervical CS-SCA is smaller in PMS than in RRMS (mean 57.0 vs 61.0 mm2, P = 0.02 at C2/C3, and mean 58.8 vs 63.4 mm2, P = 0.007 at C3/C4). (As predictive factor).
	29 Progressive MS	49.3 ± 12.2	16.6 ±8.7			
Bernitsas et al. 2015—C/S [56]	93 RRMS	39.3 ± 7.9	9.3 ± 3.3	3 T	Cross sectional area of cervical cord at C2 vertebral level	**Disability:** There was a correlation between CSA-C2 and EDSS (r = -0.75, P<0.0001). CSA-C2 was a predictor of disability independent of disease duration, and phenotype. Sub-group analysis showed a modest inverse relationship between the CSA-C2 and EDSS in the RRMS (-0.38, p = 0.0004) and progressive groups (0.4, p = 0.0021) **Progression:** CSA-C2 volume loss was more prominent in PMS compared to RRMS (68.6 ± 7.4mm2 vs. 87.3 ±8.4 mm2, p<0.0001), consistent with the neurological disability of both.
	57 PMS	44.5 ± 8.3	14.4 ± 4.5			
Bonacchi, et al. 2020—C/S [57]	58 RRMS	42.0 ± 10.0	8 (2–16) *	3 T	Cervical spinal cord	**Disability:** CSC GM-CSA is predictor of EDSS in PMS (R2 = 0.44) and RRMS (R2 = 0.51). RRMS: EDSS is associated with CSC global and regional normalized T2 lesion volume. (P values ranged from 0.02 to 0.002, except for the dorsal column, with P> 0.05) PMS: EDSS is associated with CSC GM (P = 0.003) and WM atrophy (P = 0.02). **Progression:** CSC-GM atrophy (CSA <11.1 mm2) was defined as a precise predicting factor for progressive phenotype.
	62 PMS	50.0 ± 10.0	18 (9–24) *			
	30 HC	43.0 ± 14.0	NA			
Rocca et al. 2013—C/S [71]	15 CIS	29.1 ± 7.7	0.04 (0.01- 0.08)	1.5 T	Cervical spinal cord atrophy and lesion	**Disability:** SC atrophy at C1/C2 had correlations with the pyramidal FS score in SPMS (r = −0.91, p<0.001 uncorrected) and PPMS (r = −0.89, p<0.001 uncorrected) and with EDSS in PPMS (r = −0.68, p<0.001 uncorrected). **Progression:** PPMS had significant cord atrophy.
	15 RRMS	39.2 ± 11.4	4.3 (1–10)			
	15 PPMS	43.9 ± 7.0	22.6 (15–39)			
	13 SPMS	47.6 ± 8.7	16.8 (5–34)			
	19 BMS	43.9 ± 11.1	6.7 (1–14)			
	31 HC	39.9 ± 12.6	NA			
Tsagkas et al. 2018—C [58]	180 RRMS	41.5 ± 10.1	11.4 ± 8.4	1.5 T	Spinal cord volume loss	**Disability:** SCV loss was a strong predictor of EDSS score worsening (p <0.05). In RRMS and SPMS, mean annual rate of spinal cord volume loss was identified as the strongest prediction factor of mean annual EDSS alteration. **Progression:** The mentioned predictive role for spinal cord volume loss in the previous column is stronger in SPMS than RRMS.
	51 SPMS	55.4 ± 7.6	191. ± 9.7			
Tsagkas et al. 2019—C [60]	12 PPMS	46.7 ± 6.6	8.6 ± 7.1	1.5 T	Upper cervical Spinal cord volume	**Disability:** Both PPMS and RRMS were associated with average EDSS over years, but only PPMS was associated with EDSS increase over time. **Progression:** Spinal cord volume loss was higher in PPMS compared to SPMS (p = 0.066) and RRMS (p < 0.01) over time.
	24 RRMS	48.2 ± 9.6	8.9 ± 5.9			
	24 SPMS	50.6 ± 7.4	12.3 ± 6.6			
Lukas et al. 2015—C [59]	256 RRMS	41.1 (35–48.1) *	8 (4–15) *	1.5 T	Spinal cord atrophy and lesion	**Disability:** Baseline aUCCA and the number of SC segments affected by T2-lesions were the most associated MRI markers indicative of EDSS worsening. **Progression:** All MS types had reduction of UCCA over 2 years. The annualized 24-month reduction rate of UCCA was higher in SPMS than in RRMS (p = 0.019) but did not discriminate PPMS from either RRMS or SPMS. Baseline UCCA was lower in SPMS than PPMS and RRMS. SC lesion numbers and SC affected segment numbers were higher in SPMS than PPMS and RRMS (P≤ 0.01). (SPMS was worse than other types).
	73 SPMS	54.2 (48–59.4) *	18 (12.5–26) *			
	23 PPMS	49 (44.8–56) *	6 (4–13) *			
Bischof et al. 2022—C [70]	147 RRMS remained stable	41 (18) *	5 (10) *	3 T	Cervical cord area at C1 level (C1A)	**Disability:** No association was found between C1A atrophy rates and baseline EDSS during the period of pre conversion (0.16%/year,0.50 to 0.17, p = 0.334). **Progression:** Patients who converted to SPMS indicated faster cord atrophy (-2.19% per year) compared to their RRMS matches (-0.88% per year) at least 4 years before conversion. The rate of cord atrophy was decreased after conversion (-1.63%/year, p = 0.010). Each 1% faster spinal cord atrophy rate was associated with 53% and 69% shorter time to SPMS conversion and silent progression, respectively.
	159 RRMS with silent progression	41 (13) *	6 (10) *			
	47 SPMS	47 (14) *	17 (14) *			
	80 HC	41 (18) *	NA			
Haider et al. 2021—C [34]	21 CIS	60.5 ± 7.1	30.7 ± 1	3 T	Cervical spinal cord volume	**Disability**: Cervical cord volume was associated with EDSS (β: –0.27, 95% CI: –0.421 to –0.109).
	27 RRMS	60.6 ± 6.4	30.9 ± 1			
	15 SPMS	61.9 ± 6.7	30.8 ± 0.9			
Tsagkas et al. 2021—C [40]	140 RRMS	43.8 ± 10.2	14.0 ± 8.7	1.5 T	Atrophy of spinal cord	**Disability**: SPMS: Higher CSC AVCR was associated with EDSS (Mean yearly increase of log [EDSS]of 0.024 ± 5.2 × 10–3, p = 6.7 ×10–5) and future T25FWT (Mean yearly decrease of 1/T25fwt of-4.2 × 10–3 ± 1.7 × 10–4; p = 0.087) worsening over time.
	43 SPMS	55.0 ± 8.8	21.3 ± 9.2			
Yaldizli et al. 2010—C [67]	169 MS (145 RRMS, 24 SPMS)	42.0 ± 11.3	10.9 ± 8.8	1.5 T	Corpus callosum index	**Disability:** Corpus callosum index as a correlate of brain atrophy, was associated with disability progression. Although, it was not a long-term independent predictor. CCI at diagnosis was 0.345 ± 0.04 and correlated with EDSS at diagnosis. After a follow-up of 7.1 ±6.4 years, last EDSS correlated with CCI at diagnosis and last MRI (p = 0.002; r = 0.283 and p<0.001; r = 0.301 respectively) **Progression:** 24 patients had secondary progression with lower corpus callosum index values at diagnosis compared to those remained unchanged (0.308 ± 0.08 in SPMS vs.0.353 ± 0.06 in RRMS, p = 0.003). Corpus callosum index decrease in SPMS was two times more than RRMS (p = 0.04).
Petracca et al. 2020—C/S [49]	13 RRMS	46.8 ± 11.2	14.9 ± 8.5	3 T	Streamline density and focal lesions in corpus callosum sub regions	**Disability:** Corpus callosum density was identified as an independent predictor of 9-HPT (β = -0.327, p = 0.018), T25FWT (β = -0.357, p = 0.021) and EDSS (β = -0.328, p = 0.018). Corpus callosum damage was a predictor of ambulation performance, global disability and manual dexterity. **Progression:** Streamline density decrease was distinguished in SPMS in all Corpus callosum sub-regions, in PPMS in posterior and mid-posterior corpus callosum and in RRMS, only in posterior corpus callosum. **Cognition:** Corpus callosum density was independent predictor of BVMT (β = 0.344, p = 0.023).
	20 SPMS	55.3 ± 8.4	23.0 ± 13.6			
	22 PPMS	52.3 ± 9.7	8.9 ± 5.2			
	24 HC	46.4 ± 10.5	NA			
Uher et al. 2019—C [50]	386 CIS	33.8 ± 9.0	3.0 ± 5.2	1.5 T	Corpus callosum volume	**Disability**: All patients with annualized percent corpus callosum volume change (cut-off < -0.39%) had higher EDSSAAC (p ≤ 0.001–0.035), except for CIS (p = 0.09–0.29).
	964 RRMS	35.3 ± 8.5	8.2 ± 6.5			
	63 SPMS	40.9 ± 9.1	12.6 ± 6.8			
	58 HC	37.5 ± 9.1	NA			
D’ Ambrosio et al. 2017—C/S [72]	52 RRMS	43.3 ± 11.2	8.2 (0–34) *	NA	Cerebellar subregions	**Disability:** Anterior cerebellar volume (lobules I—V) was identified as an independent predicting factor for EDSS and 9-HPT performance. **Progression:** Lower cerebellar volumes were observed in SPMS compared to BMS and RRMS patients (total and anterior cerebellar volume). **Cognition:** Cognitive performance (SDMT and PASAT scores) showed correlations with lower cerebellar volumes, prominently posterior lobe (lobules VI—X).
	20 BMS	42.6 ± 7.8	18.4 (15–26) *			
	23 SPMS	51.9 ± 9.1	19.6 (3–42) *			
	32 HC	39.6 ± 8.4	NA			
Parmar et al. 2022—C [73]	125 RRMS	44.7 ± 10.9	14.5 ± 10.9	1.5 T	Cerebellar volume	**Disability:** In RRMS, cerebellar volumes significantly predicted average EDSS, T25FWT and 9HPT. Atrophy of motor-related lobules (IV-VI + VIII) significantly predicted future worsening of non-dominant hand 9HPT. **Progression:** SPMS patients showed faster volume loss of posterior superior lobe compared to RRMS. **Cognition:** In RRMS, cerebellar volumes predicted SDMT. In SPMS, the atrophy rate of the posterior superior lobe (VI + Crus I) predicted future PASAT performance worsening.
	38 SPMS	55.1 ± 8.8	21.5 ± 9.7			
Varog˘lu et al. 2010—C/S [74]	14 RRMS	29.0 ± 11.0	2.93 ± 2.95	1.5 T	Cerebellar volume	**Disability:** Cerebellum volume and EDSS showed significant correlations in both RRMS and SPMS. **Progression:** The mean cerebellum volume was decreased in all MS patients (RRMS and SPMS together) compared to controls (129. ± 4.79 cm3, p = 0.004) and (153.4 ± 6.55 cm3, respectively; p < 0.001). The mean cerebellum volume in RRMS was higher (137 ± 5.42 cm3) than SPMS (122 ± 4.34 cm3) (p< 0.001).
	13 SPMS	38.0 ± 11.0	8.61 ± 3.33			
	26 HC	33.0 ± 8.0	NA			
Calabrese et al. 2010—C/S [75]	38 CIS	37.0 ± 8.5	0.4 ± 0.4	NA	Cerebellar cortical lesions and volume	**Disability:** The CCV (β = -0.601, p<0.001) and the cerebellar CL volume (β = 0.512, p<0.001) were the best predictors of cerebellar disability. CCV was identified as independent predictor of EDSS (b = -0.339, p = 0.011). **Progression:** Lowest CCV values were observed in PPMS. All MS subgroups had high reduction in CCV, except for CIS. Significantly higher number and volume of cerebellar CL were observed in SPMS and PPMS compared to RRMS and CIS (p<0.001 for both comparisons).
	35 RRMS	37.8 ± 7.5	6.8 ± 6.5			
	27 SPMS	43.6 ± 9.2	10.5 ± 7.4			
	25 PPMS	45.5 ± 6.2	8.4 ± 6.4			
	32 HC	35.9 ± 7.5	NA			
Petracca et al. 2022—C [83]	838 RRMS	37.7 ± 9.6	4.2 ± 5.5	NA	Cerebellar lesions number	**Disability**: Shorter 9HPT deterioration time was associated with anterior cerebellar volume (p = 0.0444) and higher cerebellar T2 lesions volume (HR = 2.211, p = 0.0002). CDP showed associations with volume and number of cerebellar Gd+ lesions (p = 0.0389 and p = 0.0223, respectively).
Favaretto et al. 2016—C/S [76]	10 CIS	30.0 ± 7.2	0.9 ± 0.6	3 T	Cerebellar cortical lesions	**Disability:** Cerebellar CL number was highly correlated with EDSS in both Double Inversion Recovery (r = 0.69, p<0.0001) and Phase Sensitive Inversion Recovery (r = 0.72, p<0.0001). **Progression:** CL was observed in 26 patients by Double Inversion Recovery and in 31 by Phase Sensitive Inversion Recovery, and their number increased from CIS/eRRMS to SPMS (p = 0.001).
	24 RRMS	40.9 ± 7.2	9.6 ± 7.0			
	6 SPMS	44.5 ± 9.8	20.2 ± 10.9			
Trufanov et al. 2021—C/S [51]	40 RRMS	31.7 ± 5.9	2.3 ±1.5	3 T	Thalamus nuclei volumes	**Disability:** EDSS had a negative correlation with the volumes of left pulvinar nuclei and the medial nucleus of right pulvinar. **Progression:** Significant differences were seen between RRMS and SPMS, in volumes of left side medial and lateral geniculate nuclei, lateral dorsal and anterior ventral nuclei. MR-morphometry of dominant deep thalamic nuclei were considered as a key predictor of MS progression. **Cognition:** Both sections (verbal and written) of the SMDT test showed moderate to strong correlations with the nuclei of thalamus, prominently with those on left side (r > 0.4). Written section of SDMT had the highest correlation with the left ventral anterior nucleus (r = 0.71).
	28 SPMS	33.3 ±5.7	5.5 ±4.4			
	10 HC	NA	NA			
Azevedo et al. 2018—C [52]	520 MS (90 CIS, 392 RRMS, 38 SPMS)	42.7 ± 9.8	9.2 ± 8.6	3 T	Thalamic volume	**Disability:** Thalamic volume reduction inversely associated with increase of EDSS (r = -0.29, CI: -0.21, -0.37) p<0.01), 9-HPT (r = -0.37, CI: -0.30, -0.45, p<0.01) and timed 25-FWT (r = -0.25, CI: -0.16, -.32, p<0.01). **Cognition:** Thalamic volume reduction associated with decrease of MSFC (r = 0.32, CI: 0.24, 0.40, p<0.01) and PASAT (r = 0.15, CI: 0.06, 0.23, p<0.01).
	81 HC	41.1 ± 9.7	NA			
Rocca et al. 2010—C/S [68]	20 CIS	28.2 ± 4.9	NA	1.5 T	Thalamic lesions volume	**Progression:** SPMS patients has significantly higher baseline mean T1 lesion volume and higher change of volume compared to RRMS (P < .001 and .002, respectively).
	34 RRMS	32.7 ± 8.4	7 (2–25) *			
	19 SPMS	40.5 ± 10.6	8 (3–23) *			
	13 HC	33.3	NA			
Louapre et al. 2018—C/S [38]	10 Early RRMS	38.3 ± 10.5	2.8 ± 1.0	3 T- 7 T	Thalamic lesions volume	**Progression**: Higher thalamic lesion volume was observed in SPMS compared to RRMS (0.16 cm3 vs 0.01 cm3). The most decreased thalamic volume was found in SPMS subjects.
	18 RRMS	44.3 ± 7.6	11.4 ± 4.2			
	13 SPMS	45.5 ± 8.1	18.5 ± 8.4			
	17 HC	39.3 ± 8.8	NA			
Magon et al. 2020—C [53]	179 RRMS	41.4 ± 10.2	11.3 ± 8.3	1.5 T	Volume loss in thalamic subnuclei	**Disability:** EDSS change was associated with anterior and ventral anterior (in MS and RRMS), and pulvinar (in MS) nucleus volume loss. Annual rates of thalamus and ventral lateral nucleus volume loss were predictive of disability worsening. **Progression:** Significant predictors of disease progression were baseline volume and annual rate of ventral lateral nucleus volume loss. Every 1% increase of the annual rate of volume loss was associated with a 20% higher risk of disease progression in the following year.
	50 SPMS	55.3 ± 7.7	18.7 ± 9.6			
Burgetova et al. 2017—C/S [54]	80 RRMS	46.9 ± 7.0	12.4 ± 10.7	1.5 T	Thalamic iron content, lesion load, brain parenchymal fraction	**Disability:** EDSS was negatively associated with thalamic iron. **Progression:** RRMS patients had significantly lower regional susceptibility in thalamus compared to PPMS group (P = 0.007).
	40 EDSS matched RRMS	48.6 ± 7.0	13.2 ± 11.0			
	24 PPMS	47.4 ± 6.8	7.7 ± 3.3			
	20 HC	48.0 ± 7.3	NA			
Tavazzi et al. 2020—C/S [64]	20 CIS	42.6 ± 10.7	5.1 ± 5.7	3 T	Thalamic volume	**Progression**: RRMS patients had significantly higher percentage of thalamic volume change compared to PMS (- 6.6% vs -4.4%, p = 0.029).
	85 RRMS	44.6 ± 10.8	13.3 ± 8.9			
	42 PMS	56.1 ± 6.3	22.5 ± 10.5			
Eijlers et al. 2018—C [77]	168 Cognitively Stable (144 RRMS, 15 SPMS, 9 PPMS)	46.77 ± 11.02	14.2 ± 8.3	3 T	Thalamic radiations	**Cognition**: Anterior thalamic radiation integrity is as a significant predictor for cognitive decline (Nagelkerke R2 = 0.35, P<0.01).
	66 Cognitively Declining (38 RRMS, 18 SPMS, 10 PPMS)	49.77 ± 10.80	15.9 ± 8.4			
	60 HC	46.45 ± 9.91	NA			
Pinter et al. 2015—C/S [79]	17 CIS	33.1 ± 9.1	0.4 ± 0.8	3 T	Thalamic volume	**Cognition**: Normalized thalamic volume strongly predicted memory function in patients.
	47 RRMS	35.8 ± 10.5	9.0 ± 8.7			
	5 SPMS	41.8 ± 9.3	11.3 ± 6.0			
Uher et al. 2019—C [50]	386 CIS	33.8 ± 9.0	3.0 ± 5.2	1.5 T	Thalamic volume	**Disability**: Patients with thalamic volume loss (cut-off < -0.56) had higher EDSSAAC (p ≤ 0.001–0.035).
	964 RRMS	35.3 ± 8.5	8.2 ± 6.5			
	63 SPMS	40.9 ± 9.1	12.6 ± 6.8			
	58 HC	37.5 ± 9.1	NA			
Tsagkas et al. 2021—C [40]	140 RRMS	43.8 ± 10.2	14.0 ± 8.7	1.5 T	Thalamic volume	**Disability**: Lower baseline thalamic volumes and higher future T25fwt deterioration were significantly associated in RRMS patients (mean yearly decrease of 1/T25fwt of -6.4 × 10–4±3.6 ×10–4, p = 0.095).
	43 SPMS	55.0 ± 8.8	21.3 ± 9.2			
Sacco et al. 2015—C/S [78]	26 Cognitively Preserved RRMS	40.0 ± 5.8	12.0 ± 7.0	3 T	Hippocampal volume	**Progression:** Significant atrophy of both hippocampus, WM and GM was observed in RRMS compared to HC (p = 0.001). **Cognition:** Atrophy of left hippocampus and WM had correlations with verbal memory performances in RRMS. In the CI subgroup, verbal memory tests significantly correlated with atrophy of left hippocampus [LTS (r = 0.46; p = 0.04), CLTR (r = 0.55; p = 0.01)].
	20 Cognitively impaired RRMS	39.1 ± 9.8	11.3 ± 6.1			
	25 HC	36.3 ± 9.2	NA			
Wen et al. 2017—C/S [80]	32 RRMS	54.2 ± 9.7	NA	3 T	Limbic system volume loss and tissue integrity	**Disability:** Reduced R2t of right amygdala correlated with worse EDSS scores (r = -0.29, p = 0.05). **Progression:** Both hippocampus and amygdala in SPMS had reduced R2t and NV compared to HC, except R2t in left amygdala. SPMS had reduced R2t of right amygdala and NV of both hippocampi compared to RRMS. PPMS patients had smaller NV in both hippocampi. **Cognition:** There was a moderate correlation of reduced R2t of the right hippocampus and amygdala with deteriorated SDMT and in left amygdala with worse performance on the 2s PASAT. Smaller NV of hippocampus and amygdala of each sides had moderate correlation with worse SDMT.
	32 SPMS	57.1 ± 9.5	NA			
	16 PPMS	55.3 ± 9.3	NA			
	31 HC	49.5 ± 15.9	NA			
Dwyer et al. 2018—C [61]	18 CIS	44.8 ± 11.0	3.9 ± 3.8	3 T	Atrophied lesion volume	**Disability:** Atrophied lesion volume had significant associations with disability progression (EDSS change) and follow-up 9HPT in both RRMS and PMS. **Progression:** Highest lesion atrophy was observed in PMS patients (P = .02).
	126 RRMS	43.8 ± 11.1	12.3 ± 8.4			
	48 PMS	55.5 ± 7.9	22.8 ± 10.3			
Tavazzi et al. 2020—C/S [64]	20 CIS	42.6 ± 10.7	5.1 ± 5.7	3 T	Atrophied lesion volume	**Progression:** Atrophied T2-LV of brain predicted worsening over time (p = .007). **Cognition:** In the progressive group, atrophied T2-LV was associated with follow-up SDMT (p = 0.003).
	85 RRMS	44.6 ± 10.8	13.3 ± 8.9			
	42 PMS	56.1 ± 6.3	22.5 ± 10.5			
Kizlaitienė et al. 2017—C/S [66]	43 RRMS	33.6 ± 9.2	7.5 ± 5.7	1.5 T	Brain atrophy (11 linear MRI measures and 7 indexes)	**Progression:** Significant discriminative MRI atrophy measurements higher in SPMS vs. RRMS are as follows: Frontal atrophy Index, EVANS index, Huckman Index, Bicaudatus Index, Width of third ventricle For differentiating RRMS from SPMS Remission-Progression Index formula can be used: Remission-Progression Index = (RAVLT 1–5 SUM + DSST)/Huckman Index **Cognition:** Brain atrophy had correlations with impairment of verbal memory and other neurocognitive symptoms. Correlation was found in bicaudatus ratio: with DSST and RAVLT1-5 SUM and width of the third ventricle. Significant discriminative Cognitive test results higher in RRMS vs. SPMS are as followed: RAVLT 1–5 SUM, DSST, DSB, FPT, ROCFT-copy, LFT-D, LFT-A, LFT-S, CATflT, IST, Story, WPA-1, WPA-2, TMTA and TMTB tests
	45 SPMS	47.8 ± 7.7	18.5 ± 7.6			
Uher et al. 2019—C [50]	386 CIS	33.8 ± 9.0	3.0 ± 5.2	1.5 T	Whole brain volume, gray matter volume	**Disability**: Patients with BVL (cut-off < -0.34%), GMVL (cut-off < -0.49), had higher EDSSAAC (p≤ 0.001–0.035), except for GMVL cut-offs in the RRMS cohort (p = 0.09–0.29).
	964 RRMS	35.3 ± 8.5	8.2 ± 6.5			
	63 SPMS	40.9 ± 9.1	12.6 ± 6.8			
	58 HC	37.5 ± 9.1	NA			
Ajitomi et al. 2022—C/S [44]	69 RRMS	39.2 ± 8.3	8 (4.2–13.3) *	1.5 T	Third & lateral ventricle width, whole-brain volume	**Disability**: EDSS had significant correlation with whole-brain volume (rho = -0.52, p <0.0001), Bicaudate ratio and width of third and lateral ventricle (stronger).
	16 PMS	46.9 ± 9.4	17.7 (9.3–25) *			
Lukas et al. 2010—C [65]	25 MS (No progression)	33 (28–38) *	1.4 (0.5–3.6) *	1 T	Annualized percentage ventricular volume change	**Disability:** There was a correlation between annual EDSS change over the 5.5-year follow-up and aPVVC. **Progression:** Patients having progression over 5.5 years had higher aPVVC within first 2 years compared with non-progressive patients (4.76%/y) compared with patients without progression (3.23% /year, p = 0.02). Each 1% increase in aPVVC every year, increases the odds of progression 1.17 times.
	29 MS (Progression)	39 (31–47) *	1.3 (0.5–3.3) *			
Rocca et al. 2013—C/S [71]	15 CIS	29.1 ± 7.7	0.04 (0.01- 0.08) *	1.5 T	Brain volume	**Progression**: Compared to RRMS and BMS, SPMS had lower NBV (p ranging from 0.03 to <0.001). They also had lower NBV than PPMS (p = 0.001).
	15 RRMS	39.2 ± 11.4	4.3 (1–10) *			
	15 PPMS	43.9 ± 7.0	22.6 (15–39) *			
	13 SPMS	47.6 ± 8.7	16.8 (5–34) *			
	19 BMS	43.9 ± 11.1	6.7 (1–14) *			
	31 HC	39.9 ± 12.6	NA			
Popescu et al. 2013—C [45]	18 CIS	29 (24–34) *	0 (0–0) *	1 T- 1.5 T	Brain atrophy and Lesion volume	**Disability:** Lesion volume change and central atrophy were predictors of EDSS in all patients and also predictors of EDSS and MSSS in ROMS group. Lesion volume at 1 year was a predictor of EDSS in RRMS and ROMS group. Baseline brain volume predicted EDSS in the CIS group. Whole brain atrophy was a predictor of EDSS and MSSS in PPMS group. **Progression:** There was an association between central atrophy and clinical progression over 5.5 years, in early RRMS.
	97 RRMS	35 (29–40) *	5 (2–8) *			
	69 SPMS	46 (40–52) *	11 (8–17) *			
	77 PPMS	52 (47–58) *	10 (6–13) *			
Moodie et al. 2012—C [46]	84 MS (57 RRMS, 20 SPMS, 7 PPMS)	42.6 ± 8.6	NA	1.5 T	Whole brain atrophy, T2 & T1 lesions volume	**Disability**: There was an association between lower baseline T2LV and EDSS worsening. Lower baseline MRI disease severity (for MRDSS and other individual MRI markers) predicted EDSS worsening.
Preziosa et al. 2022—C [47]	39 RRMS remained RRMS	36.1 ± 9.7	9.2 ± 5.4	3 T	Slowly Expanding Lesions	**Disability:** T2 hyperintense lesion volume, lower NBV, presence of ≥ 4SELs, a higher proportion of lesions defined as SELs were associated with EDSS progression at 9.1-year follow-up. Higher PBVC between year 2 and baseline was associated with EDSS score worsening. Higher proportion of SELs among baseline lesions was independent predictor of EDSS worsening (C-index = 0.892). **Progression:** T2 hyperintense lesion number and volume and lower NBV had associations with SPMS conversion over 9.1 years.
	13 RRMS converted to SPMS	39.0 ± 9.5	11.7 ± 9.2			
Pinter et al. 2015—C/S [79]	17 CIS	33.1 ± 9.1	0.4 ± 0.8	3 T	T2 lesion load	**Cognition**: T2-LL was a negative predicting factor for memory function, cognitive efficiency (βj = -0.38; p <0.001) and overall cognition (βj = -0.32; p < 0.001).
	47 RRMS	35.8 ± 10.5	9.0 ± 8.7			
	5 SPMS	41.8 ± 9.3	11.3 ± 6.0			
Filli et al. 2012—C/S [84]	106 RRMS Center 1	43.1 ± 10.7	10 (12.3) *	1.5 T	White matter lesion	**Progression**: SPMS was associated with higher regional probability of T1 (not T2) hypointense lesions in the callosal body, the corticospinal tract, and other tracts close to lateral ventricles compared to RRMS (p ≤ 0.03).
	103 RRMS Center 2	43.6 ± 9.3	8 (9) *			
	31 SPMS Center 1	53.4 ± 8.2	19 (14) *			
	31 SPMS Center 2	52.1 ± 7.4	19 (13) *			
Kincses et al. 2011—C [43]	26 CIS	36.0 ± 10.4	0.23 ± 5.5	3 T	Lesion Probability Mapping	**Disability**: EDSS had correlations with lesion probability in the periventricular WM at left frontal horn and around posterior horns.
	89 RRMS	35.2 ± 9.4	6.68 ± 7.6			
	6 SPMS	43.0 ± 8.7	10.3 ± 7.4			
Mostert et al. 2010—C [48]	96 RRMS	34 (27–41) *	2 (0–7) *	1 T- 1.5 T	T2 Lesion load and number	**Disability:** T2 LL and T2 lesion number had correlations with MSSS in RRMS. **Progression:** Higher T2 lesions number and load and confluent lesions increased disability progression risk in RRMS.
	35 SPMS	41 (32–49) *	10 (3–21) *			
	55 PPMS	48 (39–55) *	4 (2–9) *			
Papadopoulo u et al. 2013—C/S [32]	65 CIS and RRMS	49.1 ± 1.85	17.4 ± 9.4	1.5 T	White matter lesion volume	**Disability:** WMLV correlated with EDSS (r = .290, p = 0.005). **Cognition:** WM lesion volume had stronger correlations with the cognitive tests (PASAT (r = −0.361, p = 0.001), SDMT (r = 0.585, p<0.001)) compared to CL volume.
	26 SPMS and PPMS					
Calabrese et al. 2010—C/S [37]	76 RRMS	34.2	4.8	1.5 T	White matter lesion volume	**Disability**: Baseline T2 WMLV independently predicted EDSS progression in SPMS.
	31 SPMS	41.1	11.6			
Louapre et al. 2018—C/S [38]	10 Early RRMS	38.3 ± 10.5	2.8 ± 1.0	3 T- 7 T	White matter lesion volume	**Progression**: WMLV was higher in SPMS than other MS subgroups (SPMS = 13.4 vs. RRMS = 2.5 vs. early RR = 1.7).
	18 RRMS	44.3 ± 7.6	11.4 ± 4.2			
	13 SPMS	45.5 ± 8.1	18.5 ± 8.4			
	17 HC	39.3 ± 8.8	NA			
Enzinger et al. 2011—C/S [85]	62 RRMS remained RRMS	33.9 ± 8.7	5.7 ± 6.2	1.5 T	Lesion load	**Progression**: Converters to SPMS had unchanged T2 lesion load and doubled T1 lesion load and black hole ratio from baseline to follow-up compared to those who remained RRMS.
	22 RRMS converted to SPMS	40.0 ± 9.4	8.5 ± 6.9			
Sacco et al. 2015—C/S [78]	26 Cognitively Preserved RRMS	40.0 ± 5.8	12.0 ± 7.0	3 T	White matter atrophy, lesion load	**Cognition**: Atrophy of WM had correlations with verbal memory performances in RRMS. T2-LL and volume had correlations with executive functions, processing speed, visuo-spatial memory and sustained attention performances. Verbal memory tests significantly correlated with atrophy of WM in cognitively impaired subgroup [LTS (r = 0.46; p = 0.05), CLTR (r = 0.55; p = 0.01), D-SRT (r = 0.45; p = 0.05)].
	20 Cognitively impaired RRMS	39.1 ± 9.8	11.3 ± 6.1			
	25 HC	36.3 ± 9.2	NA			
Dal-Bianco et al. 2021—C [63]	24 Iron rim lesion group	36.6 (18–53.6) *	4.7 (0.9–28) *	7 T	Iron rim lesions	**Progression**: The interaction term Time × IRL status was significant in SPMS (P = 0.034) but not RRMS (P = 0.153), implicating dissimilar volume dynamics of IRLs compared to non-IRLs in SPMS.
	9 non- iron rim lesion group	31.0 (21–62.6) *	8.3 (1.1–32) *			
Tsagkas et al. 2021—C [40]	140 RRMS	43.8 ± 10.2	14.0 ± 8.7	1.5 T	Lesion load	**Disability**: Higher lesion-load AAVC was associated with higher EDSS progression and for non-dominant hand, with higher ND9HPT progression in SPMS. In RRMS, higher lesion load AAVC was associated with higher ND9HPT progression for non-dominant hand. In SPMS, higher WM AVCR was associated with higher T25fwt progression and for nondominant hand, with higher ND9HPT progression.
	43 SPMS	55.0 ± 8.8	21.3 ± 9.2			

* These values are presented as Median (Interquartile range) or Median (Range).

** Study designs are shown as C/S: cross-sectional and C: cohort studies.

F/S (T): Field strength in Tesla unit.

**Scales and tests used for assessment of physical and cognitive disability and progression in MS patients:** EDSS: Expanded Disability Status Scale; SDMT: Symbol Digit Modalities Test; PASAT: Paced Auditory Serial Addition Test; MMSE: Mini-Mental State Examination; RCPM: Raven’s Colored Progressive Matrices; RBMT: Rivermead Behavioral Memory Test; SPS: Standardized profile score; SS: Screening scores; TMT: Trail Making Test; WF: Word fluency; T25FWT: Timed 25-foot walk test; 9HPT: Nine-hole peg test; BVMT: Brief Visuospatial Memory Test; EDSSAAC: Annualized absolute EDSS change; MSFC: Multiple Sclerosis Functional Composite; MSSS: Multiple Sclerosis severity score; MRDSS: Magnetic resonance disease severity scale; RAVLT: Rey Auditory Verbal Learning Test; DSST: Digit Symbol Substitution Test.

**Abbreviations:** SD: Standard deviation; MS: Multiple Sclerosis; MRI: Magnetic Resonance Imaging; CIS: Clinically isolated syndrome; BMS: Benign MS; ROMS: Relapse-onset MS; RRMS: Relapsing-remitting MS; SPMS: Secondary progressive MS; PPMS: Primary progressive MS; PMS: Progressive MS; HC: Healthy controls; CL: Cortical lesion; ICL: Intra-cortical lesion; GMV: Gray matter volume; WM: White matter; WMLV: White matter lesion volume; NGMV: Normalized gray matter volume; AVCR: Annual volume change rate; DGM: Deep gray matter; NAWM: Normal appearing white matter; NV: Normalized volume; LV: Lesion volume; PVVC: Percentage ventricular volume change; SEL: Slowly expanding lesion; NBV: Normalized brain volume; IRL: Iron rim lesion; CCV: Cerebellar cortical volume; CS-SCA: Cross-sectional spinal cord area; CSA: Cross-sectional area; SC: Spinal cord; aUCCA: Annualized percentage upper cervical cord cross-sectional area change. PBVC: percentage brain volume change.

The evaluation of risk of bias was carried out for all the studies that were included in the analysis. Out of the total 26 cohort and 27 cross-sectional studies, only one study was found to have a high risk of bias [82], while the remaining studies were categorized as moderate or low. The detailed results of the quality assessment of the included studies are presented in Tables 2 and 3.

**Table 2:** JBI risk of bias assessment for cohort studies.

Author, year	Q1	Q2	Q3	Q4	Q5	Q6	Q7	Q8	Q9	Q10	Q11	% Yes	Risk
Haider et al., 2021	✕	✓	✓	✓	✓	✓	✓	✓	✓	✓	✓	91	Low
Treaba et al., 2019	✓	✓	✓	?	?	✕	✓	✓	✓	✓	✓	72	Low
Scalfari et al., 2018	✓	✓	✓	✓	✓	✕	✓	✓	✓	✓	✓	91	Low
Eijlers et al., 2018	?	✓	✓	✓	✓	✕	✓	✓	✕	?	✓	63	Moderate
Tsagkas et al., 2021	✓	✓	✓	?	?	✓	✓	✓	✓	✓	✓	81	Low
Eshaghi et al., 2018	?	✓	✓	✓	✓	?	✓	✓	?	?	✓	63	Moderate
Moccia et al., 2017	✓	✓	✓	✓	✓	✕	✓	✓	✓	✓	✓	91	Low
Kantarci et al., 2016	✕	?	✓	?	?	✓	✓	✓	?	?	✓	45	High
Tsagkas et al., 2018	✓	✓	✓	✓	✓	?	✓	✓	✓	?	✓	81	Low
Tsagkas et al., 2019	✓	✓	✓	✓	✓	✕	✓	✓	✓	✓	✓	91	Low
Lukas et al., 2015	✕	✓	✓	✓	✓	✕	✓	✕	?	✕	✓	54	Moderate
Bischof et al., 2022	✓	✓	✓	✓	✓	✓	✓	✓	✓	✓	✓	100	Low
Yaldizli et al., 2010	✓	✓	✓	?	?	✕	✓	✓	✓	✓	✓	72	Low
Uher et al., 2019	✕	✓	✓	✓	✓	✓	✓	✓	✓	?	✓	81	Low
Parmar et al., 2022	✓	✓	✓	?	?	✕	✓	✓	✓	✓	✓	72	Low
Petracca et al., 2022	✓	✓	✓	?	?	✕	✓	✓	✓	✓	✓	72	Low
Azevedo et al., 2018	✓	✓	✓	✓	?	✓	✓	✓	✓	✓	✓	91	Low
Magon et al., 2020	✓	✓	✓	✓	?	?	✓	✓	✓	✓	✓	81	Low
Dwyer et al., 2018	✓	✓	✓	✓	✓	✕	✓	?	?	✓	✓	72	Low
Lukas et al., 2010	✓	✓	✓	?	?	✕	✓	✓	✓	✓	✓	72	Low
Popescu et al., 2013	?	✓	✓	?	?	✕	✓	✓	✓	✓	✓	63	Moderate
Moodie et al., 2012	✓	✓	✓	✓	✓	✓	✓	✓	✓	✓	✓	100	Low
Preziosa et al., 2022	?	✓	✓	?	?	✕	✓	✓	✓	✓	✓	63	Moderate
Kincses et al., 2011	✓	✓	✓	✓	✓	✓	✓	✓	✓	✓	✓	100	Low
Mostert et al., 2010	✓	✓	✓	?	?	✓	✓	✓	✓	✓	✓	81	Low
Dal-Bianco et al., 2021	✓	✓	✓	✓	✓	?	✓	✓	✓	✓	✓	91	Low

**Abbreviations:** JBI: Joanna Briggs Institute, ‘✓’ indicates yes, ‘✕’ indicates no and ‘?’ indicates unclear.

**Q1.** Were the two groups similar and recruited from the same population? **Q2.** Were the exposures measured similarly to assign people to both exposed and unexposed groups? **Q3.** Was the exposure measured in a valid and reliable way? **Q4.** Were the confounding factors identified? **Q5.** Were strategies to deal with confounding factors stated? **Q6.** Were the groups/participants free of the outcome at the start of the study (or at the moment of exposure)? **Q7.** Were the outcomes measured in a valid and reliable way? **Q8.** Was the follow up time reported and sufficient to be long enough for outcomes to occur? **Q9.** Was follow up complete, and if not, were the reasons to loss to follow up described and explored? **Q10.** Were strategies to address incomplete follow uputilized? **Q11.** Was appropriate statistical analysis used?

**Note:** The risk of bias was ranked as high when the study reached up to 49% of “yes” scores, moderate when the study reached from 50 to 69% of “yes” scores, and low when the study reached more than 70% of “yes” scores.

**Table 3:** JBI risk of bias assessment for cross-sectional studies.

Author, year	Q1	Q2	Q3	Q4	Q5	Q6	Q7	Q8	% Yes	Risk
Papadopoulou et al., 2013	✕	✕	✓	✓	?	?	✓	✓	50	Moderate
Louapre et al., 2018	?	✓	✓	✓	✓	✓	✓	✓	87	Low
Matsushita et al., 2018	✕	✓	✓	✓	?	?	✓	✓	62	Moderate
Kalinin et al., 2020	✓	✓	✓	✓	✓	✓	✓	✓	100	Low
Calabrese et al., 2010, (Ref 37)	?	?	✓	✓	?	?	✓	✓	50	Moderate
Pinter et al., 2015	✕	✓	✓	✓	✓	✓	✓	✓	87	Low
Burgetova et al., 2017	✓	?	✓	✓	✓	✓	✓	✓	87	Low
Rocca et al., 2021	✓	✓	✓	✓	✓	✓	✓	✓	100	Low
Kearney et al., 2016	✕	?	✓	✓	?	?	✓	✓	50	Moderate
Nakamura et al., 2020	✓	✓	✓	✓	?	?	✓	✓	75	Low
Bernitsas et al., 2015	✕	✓	✓	✓	✕	?	✓	✓	62	Moderate
Bonacchi, et al., 2020	✓	✓	✓	✓	✓	✓	✓	✓	100	Low
Rocca et al., 2013	✓	✓	✓	✓	✓	✓	✓	✓	100	Low
Petracca et al., 2020	✕	?	✓	✓	✕	?	✓	✓	50	Moderate
D’ Ambrosio et al., 2017	✓	✓	✓	✓	✓	✓	✓	✓	100	Low
Varog˘lu et al., 2010	?	✕	✓	✓	?	?	✓	✓	50	Moderate
Calabrese et al., 2010, (Ref 75)	✕	✕	✓	✓	✓	✓	✓	✓	75	Low
Favaretto et al., 2016	?	✕	✓	✓	?	?	✓	✓	50	Moderate
Trufanov et al., 2021	?	✓	✓	✓	?	?	✓	?	50	Moderate
Rocca et al., 2010	✓	✓	✓	✓	?	✓	✓	✓	87	Low
Tavazzi et al., 2020	✓	✓	✓	✓	✓	✓	✓	✓	100	Low
Sacco et al., 2015	✓	✓	✓	✓	✓	✓	✓	✓	100	Low
Wen et al., 2017	✕	?	✓	✓	✓	✓	✓	✓	75	Low
Kizlaitienė et al., 2017	✓	✓	✓	✓	✓	✓	✓	✓	100	Low
Ajitomi et al., 2022	✓	✓	✓	✓	?	?	✓	✓	75	Low
Filli et al., 2012	✕	✓	✓	✓	?	?	✓	✓	62	Moderate
Enzinger et al., 2011	✕	✓	✓	✓	✓	✓	✓	✓	87	Low

**Abbreviations:** JBI: Joanna Briggs Institute, ‘✓’ indicates yes, ‘✕’ indicates no and ‘?’ indicates unclear.

**Q1.** Were the criteria for inclusion in the sample clearly defined? **Q2.** Were the study subjects and the setting described in detail? **Q3.** Was the exposure measured in a valid and reliable way? **Q4.** Were objective, standard criteria used for measurement of the condition? **Q5.** Were the confounding factors identified? **Q6.** Were strategies to deal with confounding factors stated? **Q7.** Were the outcomes measured in a valid and reliable way? **Q8.** Was appropriate statistical analysis used?

**Note:** The risk of bias was ranked as high when the study reached up to 49% of “yes” scores, moderate when the study reached from 50 to 69% of “yes” scores, and low when the study reached more than 70% of “yes” scores.

## Discussion

In recent years, MRI has emerged as a valuable tool for both the diagnosis and monitoring of MS. Extensive research has been conducted to identify predictive imaging biomarkers for MS, evaluating white and gray matter metrics to forecast disease progression. Despite being in use for almost four decades, MRI techniques are still evolving, and novel and classic metrics are being explored to improve the diagnostic process, treatment guidance, and prognosis. The significant volume of high-quality research conducted in this field of MS has enabled us to enhance our capability to correlate MRI scan outcomes with clinical evolution and pathological studies, and derive much-needed prognosis biomarkers from these data.

In this comprehensive review, we provided an insight into the potential of MRI markers to predict disability progression, disease progression, and cognitive decline in MS. The presence of lesions and alterations in certain structures of the CNS, including white matter and gray matter, corpus callosum, thalamus, and spinal cord, have been found to have a significant impact on disability progression in individuals with MS.

The progression and conversion of CIS or RRMS to progressive phenotypes of MS is a major concern among physicians and patients alike. Prognostic factors for MS progression have been identified, including various biomarkers and MRI parameters such as cortical lesion, gray matter volume change, whole brain atrophy, corpus callosum index, thalamic volume change, and certain spinal cord markers, including the presence of lesions in spinal cord or alterations in its cross-sectional area.

On the other hand, cognitive impairment is a significant and prevalent change that can occur during the course of MS. Unfortunately, it has not been deemed a sign or symptom of MS attacks or MS progression and has not been included in McDonald’s criteria until recently [23]. Despite its inevitability, cognitive impairment is often overlooked and not addressed with proper treatment and prophylaxis. Therefore, it is crucial to recognize the importance of cognitive impairment as a potential sign of disease progression and should be given the same level of attention as physical disability.

Multiple MRI parameters have been investigated as probable biomarkers for MS. Among these parameters, white matter lesions (WMLs) are a characteristic feature of MS, which are usually detected by contrast-enhanced MRI. Recent research has revealed that GM abnormalities manifest early in the course of the disease and predict both conversion to MS and the progressive accrual of disability [86]. Moreover, GM atrophy is more severe than WM atrophy in the early stages of the disease [87]. Total brain volume measurements hold significant clinical importance in MS diagnosis and monitoring. However, accurately measuring brain atrophy is crucial for detecting changes over short periods of time, and this is challenging in MS patients compared to healthy individuals due to smaller brain volumes [88–90]. While volumetrics and their derived measurements have shown promise as prognosis biomarkers for MS, the estimation of total brain atrophy in MS patients is challenging and can only be achieved after several years of longitudinal follow-up [91]. Therefore, utilizing brain atrophy as a prognosis biomarker at an individual level, particularly in the early stages of the disease, is difficult [92–95]. Furthermore, spinal cord volumetrics, especially in the cervical segment, have been found to exhibit higher atrophy in MS patients than healthy controls. Additionally, the atrophy rate of the spinal cord is higher than that of the total brain, and patients with PPMS experience more atrophy than those with RRMS [96]. Cortical lesions have been shown to have better prognostic value for clinical outcomes and disability progression than WMLs. Therefore, further research on the diagnosis, monitoring, and treatment of MS should consider cortical lesions as a valuable target [97–99].

## Conclusion

This review provides evidence for the predictive potential of various MRI landmarks in MS. Lesions and changes within CNS structures such as white matter, gray matter, corpus callosum, thalamus, and spinal cord serve as potential indicators for predicting the progression of disability. Various prognostic factors are linked to the progression of MS, encompassing the presence of cortical lesions, alterations in gray matter volume, whole brain atrophy, the corpus callosum index, changes in thalamic volume, and lesions or modifications in the cross-sectional area of the spinal cord. Regarding cognitive impairment in individuals with MS, dependable predictors include cortical gray matter volume, brain atrophy, characteristics of lesions (such as T2-lesion load, temporal, frontal, and cerebellar lesions, volume of white matter lesions), thalamic volume, and density of the corpus callosum. Overall, MRI appears to be a useful tool for predicting MS disability progression, progression of disease, and cognitive decline.

### Limitations and suggestions

MRI has been widely used to detect and monitor MS related abnormalities. However, its limitations in predicting disease progression have been noted. Firstly, conventional MRI measures may lack specificity in predicting disease progression. The lesions seen on MRI may not always correlate with clinical symptoms or disease progression. Secondly, conventional MRI may not detect early pathological changes in MS, especially in the absence of visible lesions. This can limit its ability to predict disease progression accurately. MS is a complex disease with various underlying mechanisms such as inflammation, neurodegeneration, and remyelination that conventional MRI may not capture entirely, limiting its predictive value. Lastly, conventional MRI primarily focuses on structural changes and may not fully reflect functional impairment or disability progression in MS patients. Therefore, while MRI is useful in detecting MS-related abnormalities, its limitations in predicting disease progression should be taken into consideration. There are several ways to address these limitations. Advanced MRI techniques, including Diffusion Tensor Imaging (DTI), Magnetization Transfer Imaging (MTI), and Functional Magnetic Resonance Imaging (fMRI), offer precise measures of MS progression by capturing microstructural changes, myelin content, and functional connectivity alterations. Moreover, quantitative MRI measures, such as brain atrophy rates, lesion volumes, and magnetization transfer ratios, provide objective biomarkers when combined with clinical data. Additionally, long-term follow-up studies with repeated MRI scans and clinical assessments can identify imaging biomarkers that better correlate with disease progression. Furthermore, combining conventional MRI with other modalities like Positron Emission Tomography (PET) or Optical Coherence Tomography (OCT) can offer a more comprehensive assessment of MS pathology and improve predictive accuracy.

## Supporting information

S1 Checklist (DOCX)

## References

[1] Belbasis L, Bellou V, Evangelou E, Ioannidis JP, Tzoulaki I. Environmental risk factors and multiple sclerosis: an umbrella review of systematic reviews and meta-analyses. Lancet Neurol. 2015;14(3):263–73. doi: 10.1016/S1474-4422(14)70267-4 25662901

[2] Sawcer S, Franklin RJ, Ban M. Multiple sclerosis genetics. Lancet Neurol. 2014;13(7):700–9. doi: 10.1016/S1474-4422(14)70041-9 24852507

[3] Ghasemi N, Razavi S, Nikzad E. Multiple Sclerosis: Pathogenesis, Symptoms, Diagnoses and Cell-Based Therapy. Cell J. 2017;19(1):1–10. doi: 10.22074/cellj.2016.4867 28367411 PMC5241505

[4] Saberi A, Ashkan M, Hatamian H, Ashraf A, Homaie Rad E, Bakhshi A, et al. Migraine headache in Multiple Sclerosis. Is more frequent among MS patients? Romanian Journal of Neurology. 2023;22(1):54–7.

[5] Katz Sand I. Classification, diagnosis, and differential diagnosis of multiple sclerosis. Curr Opin Neurol. 2015;28(3):193–205. doi: 10.1097/WCO.0000000000000206 25887774

[6] Macaron G, Ontaneda D. Diagnosis and Management of Progressive Multiple Sclerosis. Biomedicines. 2019;7(3). doi: 10.3390/biomedicines7030056 31362384 PMC6784028

[7] Montalban X. Primary progressive multiple sclerosis. Current Opinion in Neurology. 2005;18(3). 10.1097/01.wco.0000169742.37159.e9 15891409

[8] Oh J, Alikhani K, Bruno T, Devonshire V, Giacomini PS, Giuliani F, et al. Diagnosis and management of secondary-progressive multiple sclerosis: time for change. Neurodegener Dis Manag. 2019;9(6):301–17. doi: 10.2217/nmt-2019-0024 31769344

[9] Bogosian A, Morgan M, Moss-Morris R. Multiple challenges for people after transitioning to secondary progressive multiple sclerosis: a qualitative study. BMJ Open. 2019;9(3):e026421. doi: 10.1136/bmjopen-2018-026421 30852548 PMC6429969

[10] Krajnc N, Bsteh G, Berger T. Clinical and Paraclinical Biomarkers and the Hitches to Assess Conversion to Secondary Progressive Multiple Sclerosis: A Systematic Review. Front Neurol. 2021;12:666868. doi: 10.3389/fneur.2021.666868 34512500 PMC8427301

[11] Manouchehrinia A, Zhu F, Piani-Meier D, Lange M, Silva DG, Carruthers R, et al. Predicting risk of secondary progression in multiple sclerosis: A nomogram. Mult Scler. 2019;25(8):1102–12. doi: 10.1177/1352458518783667 29911467

[12] Rocca MA, Amato MP, De Stefano N, Enzinger C, Geurts JJ, Penner I-K, et al. Clinical and imaging assessment of cognitive dysfunction in multiple sclerosis. The Lancet Neurology. 2015;14(3):302–17. doi: 10.1016/S1474-4422(14)70250-9 25662900

[13] Oset M, Stasiolek M, Matysiak M. Cognitive Dysfunction in the Early Stages of Multiple Sclerosis-How Much and How Important? Curr Neurol Neurosci Rep. 2020;20(7):22. doi: 10.1007/s11910-020-01045-3 32444997 PMC7244611

[14] Chiaravalloti ND, Stojanovic-Radic J, DeLuca J. The role of speed versus working memory in predicting learning new information in multiple sclerosis. J Clin Exp Neuropsychol. 2013;35(2):180–91. doi: 10.1080/13803395.2012.760537 23350959

[15] Drew MA, Starkey NJ, Isler RB. Examining the link between information processing speed and executive functioning in multiple sclerosis. Arch Clin Neuropsychol. 2009;24(1):47–58. doi: 10.1093/arclin/acp007 19395356

[16] Genova HM, DeLuca J, Chiaravalloti N, Wylie G. The relationship between executive functioning, processing speed, and white matter integrity in multiple sclerosis. J Clin Exp Neuropsychol. 2013;35(6):631–41. doi: 10.1080/13803395.2013.806649 23777468 PMC4106447

[17] Owens EM, Denney DR, Lynch SG. Difficulties in planning among patients with multiple sclerosis: a relative consequence of deficits in information processing speed. J Int Neuropsychol Soc. 2013;19(5):613–20. doi: 10.1017/S1355617713000155 23425634

[18] DeLuca GC, Yates RL, Beale H, Morrow SA. Cognitive impairment in multiple sclerosis: clinical, radiologic and pathologic insights. Brain Pathol. 2015;25(1):79–98. doi: 10.1111/bpa.12220 25521179 PMC8029470

[19] Di Filippo M, Portaccio E, Mancini A, Calabresi P. Multiple sclerosis and cognition: synaptic failure and network dysfunction. Nat Rev Neurosci. 2018;19(10):599–609. doi: 10.1038/s41583-018-0053-9 30158590

[20] Wattjes MP, Ciccarelli O, Reich DS, Banwell B, de Stefano N, Enzinger C, et al. 2021 MAGNIMS-CMSC-NAIMS consensus recommendations on the use of MRI in patients with multiple sclerosis. Lancet Neurol. 2021;20(8):653–70. doi: 10.1016/S1474-4422(21)00095-8 34139157

[21] Heesen C, Kasper J, Segal J, Köpke S, Mühlhauser I. Decisional role preferences, risk knowledge and information interests in patients with multiple sclerosis. Mult Scler. 2004;10(6):643–50. doi: 10.1191/1352458504ms1112oa 15584489

[22] Brand J, Köpke S, Kasper J, Rahn A, Backhus I, Poettgen J, et al. Magnetic resonance imaging in multiple sclerosis—patients’ experiences, information interests and responses to an education programme. PLoS One. 2014;9(11):e113252. doi: 10.1371/journal.pone.0113252 25415501 PMC4240649

[23] Thompson AJ, Banwell BL, Barkhof F, Carroll WM, Coetzee T, Comi G, et al. Diagnosis of multiple sclerosis: 2017 revisions of the McDonald criteria. Lancet Neurol. 2018;17(2):162–73. doi: 10.1016/S1474-4422(17)30470-2 29275977

[24] Bergsland N, Horakova D, Dwyer MG, Dolezal O, Seidl ZK, Vaneckova M, et al. Subcortical and cortical gray matter atrophy in a large sample of patients with clinically isolated syndrome and early relapsing-remitting multiple sclerosis. AJNR Am J Neuroradiol. 2012;33(8):1573–8. doi: 10.3174/ajnr.A3086 22499842 PMC7966546

[25] Haider L, Simeonidou C, Steinberger G, Hametner S, Grigoriadis N, Deretzi G, et al. Multiple sclerosis deep grey matter: the relation between demyelination, neurodegeneration, inflammation and iron. J Neurol Neurosurg Psychiatry. 2014;85(12):1386–95. doi: 10.1136/jnnp-2014-307712 24899728 PMC4251183

[26] Schoonheim MM, Vigeveno RM, Rueda Lopes FC, Pouwels PJ, Polman CH, Barkhof F, et al. Sex-specific extent and severity of white matter damage in multiple sclerosis: implications for cognitive decline. Hum Brain Mapp. 2014;35(5):2348–58. doi: 10.1002/hbm.22332 23982918 PMC6869647

[27] Steenwijk MD, Daams M, Pouwels PJ, Balk LJ, Tewarie PK, Killestein J, et al. What explains gray matter atrophy in long-standing multiple sclerosis? Radiology. 2014;272(3):832–42. doi: 10.1148/radiol.14132708 24761837

[28] Moher D, Liberati A, Tetzlaff J, Altman DG. Preferred reporting items for systematic reviews and meta-analyses: the PRISMA statement. J Clin Epidemiol. 2009;62(10):1006–12. doi: 10.1016/j.jclinepi.2009.06.005 19631508

[29] The EndNote Team. EndNote. EndNote 20 ed. Philadelphia, PA: Clarivate; 2013.

[30] Moola S, Munn Z, Tufanaru C, Aromataris E, Sears K, Sfetc R, et al. Chapter 7: Systematic reviews of etiology and risk. JBI Manual for Evidence Synthesis: JBI; 2020.

[31] TIJ B. Joanna Briggs Institute reviewers’ manual. 2nd ed. Adelaide: The Joanna Briggs Institute; 2014.

[32] Papadopoulou A, Muller-Lenke N, Naegelin Y, Kalt G, Bendfeldt K, Kuster P, et al. Contribution of cortical and white matter lesions to cognitive impairment in multiple sclerosis. Mult Scler. 2013;19(10):1290–6. doi: 10.1177/1352458513475490 23459568

[33] Matsushita F, Kida H, Tabei KI, Nakano C, Matsuura K, Ii Y, et al. Clinical significance of cortical lesions in patients with multiple sclerosis: A neuropsychological and neuroimaging study. Brain Behav. 2018;8(3):e00934. doi: 10.1002/brb3.934 29541544 PMC5840446

[34] Haider L, Prados F, Chung K, Goodkin O, Kanber B, Sudre C, et al. Cortical involvement determines impairment 30 years after a clinically isolated syndrome. Brain. 2021;144(5):1384–95. doi: 10.1093/brain/awab033 33880511 PMC8219364

[35] Treaba CA, Granberg TE, Sormani MP, Herranz E, Ouellette RA, Louapre C, et al. Longitudinal Characterization of Cortical Lesion Development and Evolution in Multiple Sclerosis with 7.0-T MRI. Radiology. 2019;291(3):740–9. doi: 10.1148/radiol.2019181719 30964421 PMC6543899

[36] Kalinin I, Makshakov G, Evdoshenko E. The Impact of Intracortical Lesions on Volumes of Subcortical Structures in Multiple Sclerosis. AJNR Am J Neuroradiol. 2020;41(5):804–8. doi: 10.3174/ajnr.A6513 32381540 PMC7228180

[37] Calabrese M, Rocca MA, Atzori M, Mattisi I, Favaretto A, Perini P, et al. A 3-year magnetic resonance imaging study of cortical lesions in relapse-onset multiple sclerosis. Ann Neurol. 2010;67(3):376–83. doi: 10.1002/ana.21906 20373349

[38] Louapre C, Govindarajan ST, Gianni C, Madigan N, Sloane JA, Treaba CA, et al. Heterogeneous pathological processes account for thalamic degeneration in multiple sclerosis: Insights from 7 T imaging. Mult Scler. 2018;24(11):1433–44. 28803512 10.1177/1352458517726382

[39] Rocca MA, Valsasina P, Meani A, Gobbi C, Zecca C, Rovira A, et al. Association of Gray Matter Atrophy Patterns With Clinical Phenotype and Progression in Multiple Sclerosis. Neurology. 2021;96(11):e1561–e73. doi: 10.1212/WNL.0000000000011494 33441452

[40] Tsagkas C, Naegelin Y, Amann M, Papadopoulou A, Barro C, Chakravarty MM, et al. Central nervous system atrophy predicts future dynamics of disability progression in a real-world multiple sclerosis cohort. Eur J Neurol. 2021;28(12):4153–66. doi: 10.1111/ene.15098 34487400 PMC9292558

[41] Moccia M, Quarantelli M, Lanzillo R, Cocozza S, Carotenuto A, Carotenuto B, et al. Grey:white matter ratio at diagnosis and the risk of 10-year multiple sclerosis progression. Eur J Neurol. 2017;24(1):195–204. doi: 10.1111/ene.13183 27801535

[42] Eshaghi A, Prados F, Brownlee WJ, Altmann DR, Tur C, Cardoso MJ, et al. Deep gray matter volume loss drives disability worsening in multiple sclerosis. Ann Neurol. 2018;83(2):210–22. doi: 10.1002/ana.25145 29331092 PMC5838522

[43] Kincses ZT, Ropele S, Jenkinson M, Khalil M, Petrovic K, Loitfelder M, et al. Lesion probability mapping to explain clinical deficits and cognitive performance in multiple sclerosis. Mult Scler. 2011;17(6):681–9. doi: 10.1177/1352458510391342 21177325

[44] Ajitomi S, Fujimori J, Nakashima I. Usefulness of two-dimensional measurements for the evaluation of brain volume and disability in multiple sclerosis. Mult Scler J Exp Transl Clin. 2022;8(1):20552173211070749. doi: 10.1177/20552173211070749 35024162 PMC8743968

[45] Popescu V, Agosta F, Hulst HE, Sluimer IC, Knol DL, Sormani MP, et al. Brain atrophy and lesion load predict long term disability in multiple sclerosis. J Neurol Neurosurg Psychiatry. 2013;84(10):1082–91. doi: 10.1136/jnnp-2012-304094 23524331

[46] Moodie J, Healy BC, Buckle GJ, Gauthier SA, Glanz BI, Arora A, et al. Magnetic resonance disease severity scale (MRDSS) for patients with multiple sclerosis: a longitudinal study. J Neurol Sci. 2012;315(1–2):49–54. doi: 10.1016/j.jns.2011.11.040 22209496 PMC3319060

[47] Preziosa P, Pagani E, Meani A, Moiola L, Rodegher M, Filippi M, et al. Slowly Expanding Lesions Predict 9-Year Multiple Sclerosis Disease Progression. Neurol Neuroimmunol Neuroinflamm. 2022;9(2). doi: 10.1212/NXI.0000000000001139 35105685 PMC8808355

[48] Mostert JP, Koch MW, Steen C, Heersema DJ, De Groot JC, De Keyser J. T2 lesions and rate of progression of disability in multiple sclerosis. Eur J Neurol. 2010;17(12):1471–5. doi: 10.1111/j.1468-1331.2010.03093.x 20500805

[49] Petracca M, Schiavi S, Battocchio M, El Mendili MM, Fleysher L, Daducci A, et al. Streamline density and lesion volume reveal a postero-anterior gradient of corpus callosum damage in multiple sclerosis. Eur J Neurol. 2020;27(6):1076–82. doi: 10.1111/ene.14214 32170811

[50] Uher T, Vaneckova M, Krasensky J, Sobisek L, Tyblova M, Volna J, et al. Pathological cut-offs of global and regional brain volume loss in multiple sclerosis. Mult Scler. 2019;25(4):541–53. doi: 10.1177/1352458517742739 29143562

[51] Trufanov A, Bisaga G, Skulyabin D, Temniy A, Poplyak M, Chakchir O, et al. Thalamic nuclei degeneration in multiple sclerosis. J Clin Neurosci. 2021;89:375–80. doi: 10.1016/j.jocn.2021.05.043 34090763

[52] Azevedo CJ, Cen SY, Khadka S, Liu S, Kornak J, Shi Y, et al. Thalamic atrophy in multiple sclerosis: A magnetic resonance imaging marker of neurodegeneration throughout disease. Ann Neurol. 2018;83(2):223–34. doi: 10.1002/ana.25150 29328531 PMC6317847

[53] Magon S, Tsagkas C, Gaetano L, Patel R, Naegelin Y, Amann M, et al. Volume loss in the deep gray matter and thalamic subnuclei: a longitudinal study on disability progression in multiple sclerosis. J Neurol. 2020;267(5):1536–46. doi: 10.1007/s00415-020-09740-4 32040710

[54] Burgetova A, Dusek P, Vaneckova M, Horakova D, Langkammer C, Krasensky J, et al. Thalamic Iron Differentiates Primary-Progressive and Relapsing-Remitting Multiple Sclerosis. AJNR Am J Neuroradiol. 2017;38(6):1079–86. doi: 10.3174/ajnr.A5166 28450431 PMC7960078

[55] Nakamura Y, Liu Z, Fukumoto S, Shinoda K, Sakoda A, Matsushita T, et al. Spinal cord involvement by atrophy and associations with disability are different between multiple sclerosis and neuromyelitis optica spectrum disorder. Eur J Neurol. 2020;27(1):92–9. doi: 10.1111/ene.14038 31304648

[56] Bernitsas E, Bao F, Seraji-Bozorgzad N, Chorostecki J, Santiago C, Tselis A, et al. Spinal cord atrophy in multiple sclerosis and relationship with disability across clinical phenotypes. Mult Scler Relat Disord. 2015;4(1):47–51. doi: 10.1016/j.msard.2014.11.002 25787052

[57] Bonacchi R, Pagani E, Meani A, Cacciaguerra L, Preziosa P, De Meo E, et al. Clinical Relevance of Multiparametric MRI Assessment of Cervical Cord Damage in Multiple Sclerosis. Radiology. 2020;296(3):605–15. doi: 10.1148/radiol.2020200430 32573387

[58] Tsagkas C, Magon S, Gaetano L, Pezold S, Naegelin Y, Amann M, et al. Spinal cord volume loss: A marker of disease progression in multiple sclerosis. Neurology. 2018;91(4):e349–e58. doi: 10.1212/WNL.0000000000005853 29950437

[59] Lukas C, Knol DL, Sombekke MH, Bellenberg B, Hahn HK, Popescu V, et al. Cervical spinal cord volume loss is related to clinical disability progression in multiple sclerosis. J Neurol Neurosurg Psychiatry. 2015;86(4):410–8. doi: 10.1136/jnnp-2014-308021 24973341

[60] Tsagkas C, Magon S, Gaetano L, Pezold S, Naegelin Y, Amann M, et al. Preferential spinal cord volume loss in primary progressive multiple sclerosis. Mult Scler. 2019;25(7):947–57. doi: 10.1177/1352458518775006 29781383

[61] Dwyer MG, Bergsland N, Ramasamy DP, Jakimovski D, Weinstock-Guttman B, Zivadinov R. Atrophied Brain Lesion Volume: A New Imaging Biomarker in Multiple Sclerosis. J Neuroimaging. 2018;28(5):490–5. doi: 10.1111/jon.12527 29856910

[62] Scalfari A, Romualdi C, Nicholas RS, Mattoscio M, Magliozzi R, Morra A, et al. The cortical damage, early relapses, and onset of the progressive phase in multiple sclerosis. Neurology. 2018;90(24):e2107–e18. doi: 10.1212/WNL.0000000000005685 29769373

[63] Dal-Bianco A, Grabner G, Kronnerwetter C, Weber M, Kornek B, Kasprian G, et al. Long-term evolution of multiple sclerosis iron rim lesions in 7 T MRI. Brain. 2021;144(3):833–47. doi: 10.1093/brain/awaa436 33484118

[64] Tavazzi E, Bergsland N, Kuhle J, Jakimovski D, Ramanathan M, Maceski AM, et al. A multimodal approach to assess the validity of atrophied T2-lesion volume as an MRI marker of disease progression in multiple sclerosis. J Neurol. 2020;267(3):802–11. doi: 10.1007/s00415-019-09643-z 31768628

[65] Lukas C, Minneboo A, de Groot V, Moraal B, Knol DL, Polman CH, et al. Early central atrophy rate predicts 5 year clinical outcome in multiple sclerosis. J Neurol Neurosurg Psychiatry. 2010;81(12):1351–6. doi: 10.1136/jnnp.2009.199968 20826873

[66] Kizlaitiene R, Kaubrys G, Giedraitiene N, Ramanauskas N, Dementaviciene J. Composite Marker of Cognitive Dysfunction and Brain Atrophy is Highly Accurate in Discriminating Between Relapsing-Remitting and Secondary Progressive Multiple Sclerosis. Med Sci Monit. 2017;23:588–97. doi: 10.12659/msm.903234 28145395 PMC5301955

[67] Yaldizli O, Atefy R, Gass A, Sturm D, Glassl S, Tettenborn B, et al. Corpus callosum index and long-term disability in multiple sclerosis patients. J Neurol. 2010;257(8):1256–64. doi: 10.1007/s00415-010-5503-x 20198382

[68] Rocca MA, Mesaros S, Pagani E, Sormani MP, Comi G, Filippi M. Thalamic damage and long-term progression of disability in multiple sclerosis. Radiology. 2010;257(2):463–9. 20724544 10.1148/radiol.10100326

[69] Mirmosayyeb O, Brand S, Barzegar M, Afshari-Safavi A, Nehzat N, Shaygannejad V, et al. Clinical Characteristics and Disability Progression of Early- and Late-Onset Multiple Sclerosis Compared to Adult-Onset Multiple Sclerosis. J Clin Med. 2020;9(5). doi: 10.3390/jcm9051326 32370288 PMC7290335

[70] Bischof A, Papinutto N, Keshavan A, Rajesh A, Kirkish G, Zhang X, et al. Spinal Cord Atrophy Predicts Progressive Disease in Relapsing Multiple Sclerosis. Ann Neurol. 2022;91(2):268–81. doi: 10.1002/ana.26281 34878197 PMC8916838

[71] Rocca MA, Valsasina P, Damjanovic D, Horsfield MA, Mesaros S, Stosic-Opincal T, et al. Voxel-wise mapping of cervical cord damage in multiple sclerosis patients with different clinical phenotypes. J Neurol Neurosurg Psychiatry. 2013;84(1):35–41. doi: 10.1136/jnnp-2012-303821 23064100

[72] D’Ambrosio A, Pagani E, Riccitelli GC, Colombo B, Rodegher M, Falini A, et al. Cerebellar contribution to motor and cognitive performance in multiple sclerosis: An MRI sub-regional volumetric analysis. Mult Scler. 2017;23(9):1194–203. doi: 10.1177/1352458516674567 27760859

[73] Parmar K, Fonov VS, Naegelin Y, Amann M, Wuerfel J, Collins DL, et al. Regional Cerebellar Volume Loss Predicts Future Disability in Multiple Sclerosis Patients. Cerebellum. 2022;21(4):632–46. doi: 10.1007/s12311-021-01312-0 34417983 PMC9325849

[74] Varoglu AO, Odaci E, Gumus H, Keles ON, Unal B, Deniz O. Evaluation of patients with multiple sclerosis using a combination of morphometrical features and clinical scores. J Clin Neurosci. 2010;17(2):191–5. 20036126 10.1016/j.jocn.2009.04.023

[75] Calabrese M, Mattisi I, Rinaldi F, Favaretto A, Atzori M, Bernardi V, et al. Magnetic resonance evidence of cerebellar cortical pathology in multiple sclerosis. J Neurol Neurosurg Psychiatry. 2010;81(4):401–4. doi: 10.1136/jnnp.2009.177733 19965849

[76] Favaretto A, Lazzarotto A, Poggiali D, Rolma G, Causin F, Rinaldi F, et al. MRI-detectable cortical lesions in the cerebellum and their clinical relevance in multiple sclerosis. Mult Scler. 2016;22(4):494–501. doi: 10.1177/1352458515594043 26163070

[77] Eijlers AJC, van Geest Q, Dekker I, Steenwijk MD, Meijer KA, Hulst HE, et al. Predicting cognitive decline in multiple sclerosis: a 5-year follow-up study. Brain. 2018;141(9):2605–18. doi: 10.1093/brain/awy202 30169585

[78] Sacco R, Bisecco A, Corbo D, Della Corte M, d’Ambrosio A, Docimo R, et al. Cognitive impairment and memory disorders in relapsing-remitting multiple sclerosis: the role of white matter, gray matter and hippocampus. J Neurol. 2015;262(7):1691–7. doi: 10.1007/s00415-015-7763-y 25957638

[79] Pinter D, Khalil M, Pichler A, Langkammer C, Ropele S, Marschik PB, et al. Predictive value of different conventional and non-conventional MRI-parameters for specific domains of cognitive function in multiple sclerosis. Neuroimage Clin. 2015;7:715–20. doi: 10.1016/j.nicl.2015.02.023 25844323 PMC4375639

[80] Wen J, Yablonskiy DA, Salter A, Cross AH. Limbic system damage in MS: MRI assessment and correlations with clinical testing. PLoS One. 2017;12(11):e0187915. doi: 10.1371/journal.pone.0187915 29121642 PMC5679614

[81] Kearney H, Miszkiel KA, Yiannakas MC, Altmann DR, Ciccarelli O, Miller DH. Grey matter involvement by focal cervical spinal cord lesions is associated with progressive multiple sclerosis. Mult Scler. 2016;22(7):910–20. doi: 10.1177/1352458515604905 26432854

[82] Kantarci OH, Lebrun C, Siva A, Keegan MB, Azevedo CJ, Inglese M, et al. Primary Progressive Multiple Sclerosis Evolving From Radiologically Isolated Syndrome. Ann Neurol. 2016;79(2):288–94. doi: 10.1002/ana.24564 26599831

[83] Petracca M, Cutter G, Cocozza S, Freeman L, Kangarlu J, Margoni M, et al. Cerebellar pathology and disability worsening in relapsing-remitting multiple sclerosis: A retrospective analysis from the CombiRx trial. Eur J Neurol. 2022;29(2):515–21. doi: 10.1111/ene.15157 34695274

[84] Filli L, Hofstetter L, Kuster P, Traud S, Mueller-Lenke N, Naegelin Y, et al. Spatiotemporal distribution of white matter lesions in relapsing-remitting and secondary progressive multiple sclerosis. Mult Scler. 2012;18(11):1577–84. doi: 10.1177/1352458512442756 22495945

[85] Enzinger C, Fuchs S, Pichler A, Wallner-Blazek M, Khalil M, Langkammer C, et al. Predicting the severity of relapsing-remitting MS: the contribution of cross-sectional and short-term follow-up MRI data. Mult Scler. 2011;17(6):695–701. doi: 10.1177/1352458510394454 21228028

[86] Tiu VE, Enache I, Panea CA, Tiu C, Popescu BO. Predictive MRI Biomarkers in MS-A Critical Review. Medicina (Kaunas). 2022;58(3). doi: 10.3390/medicina58030377 35334554 PMC8949449

[87] Sastre-Garriga J, Ingle GT, Chard DT, Cercignani M, Ramió-Torrentà L, Miller DH, et al. Grey and white matter volume changes in early primary progressive multiple sclerosis: a longitudinal study. Brain. 2005;128(Pt 6):1454–60. doi: 10.1093/brain/awh498 15817511

[88] Chu R, Tauhid S, Glanz BI, Healy BC, Kim G, Oommen VV, et al. Whole Brain Volume Measured from 1.5T versus 3T MRI in Healthy Subjects and Patients with Multiple Sclerosis. J Neuroimaging. 2016;26(1):62–7. doi: 10.1111/jon.12271 26118637 PMC4755143

[89] Audoin B, Ibarrola D, Malikova I, Soulier E, Confort-Gouny S, Duong MV, et al. Onset and underpinnings of white matter atrophy at the very early stage of multiple sclerosis—a two-year longitudinal MRI/MRSI study of corpus callosum. Mult Scler. 2007;13(1):41–51. doi: 10.1177/1352458506071215 17294610

[90] Maclaren J, Han Z, Vos SB, Fischbein N, Bammer R. Reliability of brain volume measurements: a test-retest dataset. Sci Data. 2014;1:140037. doi: 10.1038/sdata.2014.37 25977792 PMC4411010

[91] Biberacher V, Schmidt P, Keshavan A, Boucard CC, Righart R, Sämann P, et al. Intra- and interscanner variability of magnetic resonance imaging based volumetry in multiple sclerosis. Neuroimage. 2016;142:188–97. doi: 10.1016/j.neuroimage.2016.07.035 27431758

[92] Fragoso YD, Willie PR, Goncalves MVM, Brooks JBB. Critical analysis on the present methods for brain volume measurements in multiple sclerosis. Arq Neuropsiquiatr. 2017;75(7):464–9. doi: 10.1590/0004-282X20170072 28746434

[93] Vidal-Jordana A, Sastre-Garriga J, Pérez-Miralles F, Tur C, Tintoré M, Horga A, et al. Early brain pseudoatrophy while on natalizumab therapy is due to white matter volume changes. Mult Scler. 2013;19(9):1175–81. doi: 10.1177/1352458512473190 23319072

[94] Rocca MA, Battaglini M, Benedict RH, De Stefano N, Geurts JJ, Henry RG, et al. Brain MRI atrophy quantification in MS: From methods to clinical application. Neurology. 2017;88(4):403–13. doi: 10.1212/WNL.0000000000003542 27986875 PMC5272969

[95] Barkhof F. Brain atrophy measurements should be used to guide therapy monitoring in MS—NO. Mult Scler. 2016;22(12):1524–6. doi: 10.1177/1352458516649452 27335096

[96] Casserly C, Seyman EE, Alcaide-Leon P, Guenette M, Lyons C, Sankar S, et al. Spinal Cord Atrophy in Multiple Sclerosis: A Systematic Review and Meta-Analysis. J Neuroimaging. 2018;28(6):556–86. doi: 10.1111/jon.12553 30102003

[97] Rinaldi F, Calabrese M, Grossi P, Puthenparampil M, Perini P, Gallo P. Cortical lesions and cognitive impairment in multiple sclerosis. Neurol Sci. 2010;31(Suppl 2):S235–7. doi: 10.1007/s10072-010-0368-4 20635113

[98] Scalfari A, Neuhaus A, Degenhardt A, Rice GP, Muraro PA, Daumer M, et al. The natural history of multiple sclerosis: a geographically based study 10: relapses and long-term disability. Brain. 2010;133(Pt 7):1914–29. doi: 10.1093/brain/awq118 20534650 PMC2892939

[99] Calabrese M, Poretto V, Favaretto A, Alessio S, Bernardi V, Romualdi C, et al. Cortical lesion load associates with progression of disability in multiple sclerosis. Brain. 2012;135(Pt 10):2952–61. doi: 10.1093/brain/aws246 23065788

